# Using Photovoice to investigate the lived experiences of family care partners of people living with dementia: What supports their well-being in a care relationship?

**DOI:** 10.3389/fpubh.2026.1825358

**Published:** 2026-07-01

**Authors:** Ieva Petkutė, Simona Karpavičiūtė, Diana Rėklaitienė

**Affiliations:** 1 Department of Couching Science, Lithuanian Sports University, Kaunas, Lithuania; 2 NGO “Socialiniai Meno Projektai”, Vilnius, Lithuania

**Keywords:** arts, care relationship and caregiver support, dementia, dementia risk reduction and health promotion, family care partners, health and well-being, Photovoice, public health and dementia policy

## Abstract

**Background:**

Dementia is one of the main global health challenge and public health priority. The aim of this study was through the Photovoice practice to investigate the lived experiences of what supports the well-being of family care partners of people living with dementia in a care relationship.

**Methods:**

Family care partners of people living with dementia took part in the study. Data was collected using questionnaire survey (before the study); photographs, stories and observational notes (during the study), as well as individual meetings and group discussions (during and after the study). Qualitative research results were analyzed using a thematic analysis approach.

**Results:**

The following themes emerged from the study—“Reconciliation and Acceptance,” “The Aspiration to Maintain One's Inner Freedom and Well-being,” “The Bond in Care,” and “The Role of Supportive-social Environment.” Creative results were presented in a public exhibition.

**Conclusions:**

It's timely to actively find ways to sustain the care provided by family care partners of people living with dementia. Recognizing informal carers as an important part of the dementia care system, and actively developing efficient informal carer health and well-being promotion strategies both at individual, family, and society levels, may counteract psychological, physical and social challenges and reduce the prevalence of stigma within the carer journey. The study findings suggest that the Photovoice is a promising tool for identifying potential support systems for the welfare of family care partners of people living with dementia, reducing the stigma and contributing to their well-being. In this study, participants had opportunities to connect with other caregivers, to be active while engaging in creative activity, to take notice and learn new things, and to share their experiences with others both in a group settings and in a public exhibition. Photovoice can be applied more broadly in research and policy-making when working with vulnerable populations.

## 1 Introduction

Someone in the world develops dementia every 3 s. There were over 55 million people worldwide living with dementia in 2020. This number will almost double every 20 years, reaching 78 million in 2030 and 139 million in 2050. Already 60% of people with dementia live in low and middle income countries, but by 2050 this will rise to 71% (1).

Dementia results from a variety of diseases and injuries that affect the brain. Alzheimer's disease is the most common form of dementia and may contribute to 60%−70% of cases. Dementia is currently the seventh leading cause of death and one of the major causes of disability and dependency among older people globally (2). Dementia mainly affects older people, although there is a growing awareness of cases that start before the age of 65 (1). Dementia among younger people is rapidly emerging as a global health concern (3).

The majority of persons living with dementia are primarily cared for by their family members at home (4). The importance of family care is evident both in terms of estimated economic impact of dementia—informal care costs account for up to 40% of the total dementia care related expenses (5)—and as a key factor in the quality within person-oriented care provision (6). In 2019, dementia cost economies globally US$ 1.3 trillion, and approximately 50% of these costs are attributable to care provided by informal carers (e.g., family members and close friends), who provide on average 5 h of care and supervision per day (2).

Overall, the progression of dementia is impacted by the personality (7), lifestyle (8) and other health conditions that the person has had throughout their life up to the onset of dementia (9). For these reasons, there's an increasing awareness of the need to provide personalized medicine and individualized support to every person who develops dementia (10), and family carers are increasingly recognized as one of the primary resources to provide such care within the long term care system (11).

Women are disproportionately affected by dementia, both directly and indirectly (2) and they make up a majority of informal caregivers (12).

The accumulation of these negative outcomes can increase caregiver burden, an experience that is described as a combination of the psychological, physical, social, and financial strains (12), and women caregivers tend to experience worse mental health outcomes than their male counterparts (13). It has been proven that the care of people living with dementia impacts health, social, and psychological well-being of their caregivers, often referred to as caregiver burden (14).

Lack of information and control over the situation and uncertainty are the most stressful stimuli, as a result, many carers experience high levels of stress on a regular basis. Stress in dementia care is expressed in different ways, such as anger outbursts, social withdrawal, anxiety, depression, exhaustion, irritability, lack of concentration, and numerous other mental and physical problems (15). The root of stress may be associated not only with the deteriorating condition of the relative, but also with the carer's capacity to take care of their own well-being (16). Caregivers providing high-intensity care reported feeling more tired, experienced high rates of sleep disturbance, and performed less physical activity (17).

Negative impact of care on person's well-being is also reinforced by prevailing attitudes toward dementia. Caring for people living with dementia is associated with significantly limited social life due to fear of others' perceptions and concerns about reactions to their loved one (18), as well as feelings of loneliness, social isolation (19, 20). Social support and caregiving related factors influence family caregivers' quality of life and caregiving self-efficacy (4). Social isolation exacerbates as dementia progresses, and so does the need for health and support, which in turn requires more time and energy, resulting in a lack of free time for the carer (21, 22).

The Federation of national Alzheimer's disease associations, which operates in partnership with the World Health Organization, states that standing on the frontline of dementia, both formal and informal carers deserve access to the tools, materials, and psychological support they need to be able to accompany people living with dementia with dignity, respect, and compassion (23). According to the authors (24), there is a need for targeted interventions to promote self-care practices that can enhance caregivers' health, including better sleep hygiene, balanced nutrition, and regular exercise. Given the high frequency of poor health related quality of life in dementia caregivers and the important recognition of its serious consequences on physical and mental health, clinicians should take into consideration effective interventions to improve health and health related quality of life for family caregivers of people living with dementia (25).

Therefore, this study, presented in this article, aimed to investigate the lived experiences of family care partners of people living with dementia to identify what supports their well-being in a care relationship through the implementation of the Photovoice practice in Lithuania, in order to better outline the future initiatives that meet their needs and promote their health and well-being.

## 2 Materials and methods

The Photovoice research project comprised two related studies involving the same cohort of participants and sharing the methodological approach—Photovoice Study I and Photovoice Study II. The research question of Photovoice Study I was “What are the challenges to the well-being of family care partners of people living with dementia in a care relationship?” and the research question of Photovoice Study II was “What supports the well-being of family care partners of people living with dementia in a care relationship” In this article, we present the findings of the Photovoice Study II. And the findings of Photovoice Study I which explores the challenges to the well-being of family care partners of people living with dementia in a care relationship could be found in the related publication.^1^

### 2.1 Study population

Participants: family care partners of people living with dementia took part in the study (Photovoice research project).

*The participants' inclusion criteria* were as follows:

- Adult living in Lithuania;
- The person is a care partner (family caregiver and relative) of a person living with dementia, and lives together with them or lives separately but visits regularly for care purposes at least four times per week;
- Care partner does not receive any salary and is not paid for the provision of daily care to a person living with dementia at their home;
- Care partner is available and agrees to take part in the Photovoice research project;
- Care partner has no previous experience of Photovoice practice.

### 2.2 Study design

Photovoice was established by Wang and Burris (26), and is a process by which people can identify, represent, and enhance their community through a specific photographic technique (27). As a practice based on the production of knowledge (26), Photovoice has three main goals: to enable people to record and reflect on their community's strengths and concerns; to promote critical dialogue and knowledge about important issues through large and small group discussion of photographs; and to reach policymakers (27). Photovoice is a participatory action research strategy by which people create and discuss photographs as a means of catalyzing personal and community change (28). As a community-based participatory research method, Photovoice contributes to an enhanced understanding of community assets and needs and to empowerment (29). Based on visual research methodology, Photovoice aims to foster social change, and it allows participants (community members) to express their lived experiences by using photography. Currently, this method is considered an analytical, proactive, and empowering methodology to work with diverse communities, supporting individuals to reflect upon their strengths and challenges, to better understand their own situation, which can then be communicated to a wider society and specifically people who make decisions that have an impact on the life of the community. In this approach, participants' expertise and wisdom are seen as values to be honored and thus voiced to a wider community or decision makers to reflect participants' perspectives on structural systems (30).

There was an open call for participation in the Photovoice research project for persons to be involved in the participant group. An open call was announced electronically via the Association “Dementia Lithuania” and partner communication channels. After the formation of the participant group, the open call for the registration was closed. The evaluation process of each application was carefully examined by the independent evaluator. After the screening of the applications, in March, the lead researcher-facilitator contacted each applicant and discussed their application in person via Zoom or video call. It was important to ensure that only persons meeting the inclusion criteria were selected to be included in the study. The study organizers offered a total of 10 places for the participants to take part in the Photovoice research project. Subsequently, 10 members of family care partners were registered to take part in the Photovoice research project.

*The study questions in the Photovoice research project were the following:* “What supports the well-being of family care partners of people living with dementia in a care relationship?” (addressed in this article) and “What are the challenges to the well-being of family care partners of people living with dementia in a care relationship?” [addressed in the related publication (see text footnote ^1^)].

*Study place and timeframe*: The Photovoice study was carried out from March to September 2024 in Lithuania. The main part of the on-site activities (Photovoice practice live trainings-discussions) took place in Kaunas city. Photography related tasks (homework) to be completed at participants' homes were assigned each week after the on-site meeting. There were also visits to each of participant's home (in three major cities, two suburban cities and two suburban villages across Lithuania), as well as online discussions and meetings held with participant group and individually via the Zoom platform.

The live Photovoice activities were offered once a week on-site for 4 weeks during the weekends (on Saturdays), with a 2-week break after the second meeting. Care partners in the participant group could choose the most convenient day and time to attend the photography activities. This day was selected and agreed upon taking into account the participants' availability to travel to the onsite meeting/training from their hometowns (to ensure the possibility of leaving their loved ones living with dementia in the care of someone else, either voluntarily or through a paid caregiver). For some participants, this was the first time requesting external help and leaving their cared-for loved ones with another person.

The photography activities (Photovoice practice) were not art therapy sessions, but arts for health activities, where participants could practice their photography skills, awaken their creativity while exploring the research question, communicate with other informal carers, share their experiences with each other, and feel part of the caregiver community.

*Timing*: The Photovoice practice on-site meetings lasted 3–4 h.

*Instructors*: The team was composed of a lead researcher-artist facilitator, a visual artist, a photographer, and an independent researcher who participated in on-site activities.

*Costs of participation in the study/Photovoice practice*: Participation in the study activities was free of charge and voluntary. Neither the study participants, the main researcher and facilitator, nor an independent researcher received financial compensation for the study.

The organizers offered to cover the following optional expenses for participants: travel costs (travel tickets or petrol) to attend the Photovoice on-site activities, and a fee to cover the cost of hiring someone to provide care while the care partner attended the four on-site meetings (maximum of 100 Eur.). During the Photovoice practice, participants were offered free cold and hot beverages and light meals/snacks on site. In addition, participants received a gift of their portraits after the study, showing whether the caregiver alone or the caregiver together with their loved one living with dementia, taken by a professional photographer at their preferred location or home.

The expenses included:

- Exhibition costs, including printing of photographs and captions, materials, and the exhibition book;
- Printing costs of photographs used during the Photovoice practice activities;
- Honorariums for the photographer (who attended the Photovoice practice activities and created the portraits) and the visual artist (who carried out photo documentation of the on-site meetings and exhibition opening, assisted with exhibition installation, and designed the exhibition book);
- Research team's travel expenses (to and from on-site meetings and participants' homes) and photographer's travel costs to participants' homes to create portraits.

All costs were covered from the project budget (see the Funding section).

*Language*: The Photovoice practice was conducted in Lithuanian, which was the native language of the participants.

*Technical equipment/tools used for the Photovoice practice activities*: Notebook, pens, markers, paper sheets, stickers; whiteboard and markers; smartphones; laptop; stable internet connection; Zoom account and the visual collaboration tool “Padlet”; voice recorders; external memory drive.

### 2.3 Measurements and instruments

#### 2.3.1 The anonymous questionnaire survey (additional data)

The research questionnaire, administered during the registration process, prior to the study (Photovoice research project), consisted of:

- Socio-demographic questions (e.g., as gender, age, place of residence, etc.);
- Questions about caregiving experience (care characteristics);
- Questions about participants' experience in photography activities, including Photovoice practice, and their motivation to take part in the study, etc (Supplementary Data 1.1.).

#### 2.3.2 The Photovoice practice

The engagement in the Photovoice practice included the following steps:

1. **Introduction** Selected research participants who agreed to be part of the study were invited to an introductory sessions to learn about the Photovoice practice, explore the goals and timeline, discuss the topic of well-being, consider ethical aspects, and understand the commitment required. The session also provided an opportunity for participants get to know each other, connect, and start building relationships. It was led by a lead facilitator-artist researcher and a photographer. Everyone could raise questions or concerns they might have about the participation in the practice.
2. **Skills development** Introductory and second on-site sessions were also focused on developing photography skills, including:

- *Familiarizing with the technical aspects of the camera*. Participants needed to be comfortable using a camera to gain confidence in representing their experiences through images. Thus, an experienced photographer was invited to guide the participants, helping them become familiar with the camera as a tool itself, and encouraging reflection on what makes a photograph better or more powerful. As preferred by the group, all participants used their mobile phones for photography. The photographer and visual artist checked the camera settings on the participants' phones to ensure the highest quality of photo resolution possible at a time, provided instructions on features such as lighting and other technical settings, and offered further guidance on experimenting and exploring these features.
- *Visual literacy development*. Participants explored how different choices in perspective, light, and framing affect the visual impact of the image. Practical exercises, such as photographing the built environment, objects and each other's portraits, supported this learning process.
- *Introduction to photography vocabulary*. The participants were introduced to the basic photography terminology to encourage the expression of insights and ideas about their own work and the work of others.
- *Introduction to photography ethics*. The participants were introduced to photography ethics, including how to responsibly photograph people, objects, and places, and the importance of obtaining consent. The lead researcher provided practical tools to help ensure that participants engage in ethical photography practices.
- *Exploration of the individual style*. Participants were encouraged to explore their own style and inner voice when making choices, to improvise, and experiment with the camera. Additional practical tasks guided participants to gradually develop awareness of their individual creative perspective on their surroundings, gain insight into what sparks their curiosity, and explore ways to express abstract ideas and emotions.
- *Connection building*. Practical tasks and discussions along the way encouraged connection building and conversations among the group members. Each person could take as many pictures as they wanted and was invited to select a limited number that, in their opinion, best represented the task. All the creative results were presented to the large group, with comments and discussions led by the photographer, facilitator, and by each participant. The task and sharing of results helped participants practice their photography skills, build community spirit, foster creativity, discover similarities among the group members, and prepare them to better manage the homework.

3. **Individual creative exploration** As a homework task for the third meeting, the participants were asked to take photos exploring their current daily life, responding to the two distinct research questions: “*As a care partner for a person living with dementia, what supports my well-being in a care relationship?*” which is presented in this article. And “*What are the challenges to the well-being of family care partners of people living with dementia in a care relationship?*” presented in the related publication (see text footnote ^1^). Participants could take as many pictures as they wished, selecting those that, in their opinion, most strongly represented the issue, to share with the group and instructors. There was a 2-week period between the second and the third meeting to respond to this task. Each participant was encouraged to take as many photographs as they liked, and through the process, pay attention to anything that is related to the research question through the lens of their physical and mental health and well-being—their feelings, thoughts, specific events or circumstances, situations, particular supports and challenges associated with their caring role.
4. **Individual support** During this period, the lead-facilitator and researcher visited each participant at their homes (from the end of April to the beginning of May) to follow up on their homework, encourage task completion, support the awakening of their creativity, and answer any questions. The pictures taken during this 2-week period had to be sent to the lead facilitator, who then printed them in paper format for the third on-site meeting.
5. **Creative results presentation** During the third on-site meeting, each participant presented their creative results. Every participant was given the opportunity to share their intimate stories that emerged from their photographs. The SHOWED technique (27) was used to facilitate the sharing and discussion of photographs. After each presentation, all other members were encouraged to express sincere appreciation for everyone's work by offering comments and asking questions that would help deepen their understanding of the photographs. If questions were posed by others in the small group, the presenter could choose whether to respond.
6. **Creating a thematic photography tree** The participants were divided into smaller groups for discussions and for jointly grouping the photographs thematically. Each small group shared their findings and themes and selected the core photographs to be used moving forward. As the photographs were discussed, some ideas emerged repeatedly. The participants then worked together as one group, discussing ideas about the photos, grouping them thematically, and narrowing down the overarching themes as well as the number of the photographs in each theme. Ultimately, the participants, together with the lead researcher-facilitator, formed a thematic photography tree. Following this, participants were invited to take additional photos if they felt the need or to re-take certain photographs if they were not fully satisfied with them. The notes taken during the discussions were recorded and transcribed. After the group work with participants, two researchers reviewed the listed themes, discussed and resolved any uncertainties, and made minor refinements to the final list. A summary report of the themes that emerged from the conversations was then drafted and presented to the group of participants, asking for their input and validation to ensure that it was reflective of the discussion. No further corrections were made.
7. **Creating titles and stories** After the fourth meeting, the lead facilitator held individual discussions with each participant via the Zoom platform to further review their photographs and titles, and to help them develop the captions—the stories behind the images. The discussions were facilitated using the SHOWED technique (27) to support sharing and conversation about the meanings related to the photographs, with some additional integrated question prompts (30) such as: What do you See here? (Describe the picture as if someone cannot see it); What is really Happening here? (Describe the actions and feelings in the picture); How does this relate to Our lives? (Describe how you feel about the picture and how your experiences are similar or different from what is shown); Why does this situation, concern or strength Exist? (Describe the underlying meaning and root causes of what is in the picture and its impacts on you and your community); What can we Do about it? (Describe actions that can be taken to solve problems or build upon strengths). Participants could ask any questions they had. The stories were recorded, transcribed, and drafted into a presentable format by the lead researcher-facilitator. They were then sent to each participant for validation and for any further input or corrections, if desired.
8. **Group validation of Photovoice creative results** The final themes, photographs, captions, and stories were validated and co-created in collaboration with the participants. All participants were invited to discuss how they wanted to share their photographs with the community and to co-create a title for the exhibition. Participants could choose the name or nickname by which they wished to be represented in the exhibition. During the exhibition planning—including selecting the photographs, creating the title, and choosing the venue—everyone in the participant group was invited to take part, and those who expressed interest actively participated in the planning process.
9. **Participants' portraits** The photographer visited the participants at their homes and created their portraits. The portraits were presented to the public as part of the exhibition. In addition, the participants received a gift of an individual copy of their portrait.
10. **Presentation to the public** The creative results—selected participants' photographs, their captions and their stories, and participants' portraits, all validated by participants—were presented to the public as a free-of-charge gallery exhibition, as part of the leading events programme in Kaunas in September 2024 and in an accompanying exhibition catalog-photography book. Exhibition and the events programme attracted a diverse audience. Several participants chose to act as guides and led a public tour, presenting their photographs and stories. This opportunity further strengthened their self-esteem and sense of empowerment.

### 2.4 Data collection

Data were collected using a questionnaire (during the registration—before the study, the first on-site meeting); Photovoice practice, photographs and stories, observational notes (during the study); individual meetings with each participant and group discussions (during the study; and after the study: after the fourth on-site meeting).

Before the questionnaire survey, participants were introduced in writing to the purpose and content of the questionnaire, the process of completing it, and the use of data. The participants were able to agree to or decline participation in the questionnaire survey, as well as to withdraw from it. If they had any questions regarding the conduct of the questionnaire survey, respondents could contact the person administering it.

The survey questionnaires were distributed to the respondents. The time to fill out the survey questionnaire was not limited, i.e., the needs and capabilities of each person were taken into account. The completed questionnaires were collected and used for the analysis of the received questionnaire data. The received questionnaire data were summarized and used for research purposes only. Participants understood the questionnaire well. There were no unanswered or incomplete questions (thus, all 10 questionnaires were included in further data analysis). The anonymity and confidentiality of the questionnaire data were ensured.

Before the study/Photovoice practice participants were provided with an information form about the study and signed an informed consent form to participate in the study and for the dissemination of the results. Before the individual meeting-discussion about the photographs (for further development of the captions), the participants were introduced orally to the purpose of the activity, the structure and process of responding and the use of data. The participants were able to agree to or decline participation in the activity, as well as to withdraw from it. If there were any questions regarding the conduct of the activity, respondents could ask the person conducting it. The time for the activity was not limited, i.e., the needs and capabilities of each person were taken into account. The activity was recorded and used for the analysis of the received data. The received data were transcribed, summarized and used for study purposes only.

The anonymity and confidentiality of individual meetings/group discussions data were ensured as agreed with participants. Observational notes regarding the study activities and discussions were taken (during the study) to ensure the quality of the process and the relevance of the study data. At all stages of the study, participants could share their experiences freely, decide what they wished to present to others, as well as validate the results and take part in planning the exhibition as well as engage in its on-site programme activities.

### 2.5 Data analysis

The qualitative data collected during process applying Photovoice methodology were further analyzed using the thematic analysis approach by Braun and Clarke (31), where the phases of thematic analysis included familiarizing with data, generating initial codes, searching for themes, reviewing the themes, defining and naming the themes, producing the report. Statistical data analysis included descriptive statistics (frequency, percentage, etc.). Survey data were processed using the statistical software IBM SPSS Statistics 22 (IBM Corporation, Armonk, NY, USA) and MS Excel 2013 (Microsoft Corporation, USA).

### 2.6 Data storage

The digital data are stored on a separate, dedicated SanDisk USB flash drive for the expected data retention period, in accordance with ethics committee regulations. The principal investigator has access to the study data. The data are protected with additional security—a password.

### 2.7 Ethical permission

The information about the ethical approval for this study is provided in the Ethical statement section.

## 3 Results

### 3.1 General characteristics of participant group

Dementia care partners (the participant group) took part in the study. Socio-demographic characteristics of participant group in the Photovoice research project showed that all participants were female (*n* = 10). Five family care partners were in age group from 40 to 54 years (40 years, *n* = 1; 42 years, *n* = 1; 45 years, *n* = 1; 51 years, *n* = 1; 54 years, *n* = 1) and four from 55 to 70 years (55 years, *n* = 1; 60 years, *n* = 1; 63 years, *n* = 1; 68 years, *n* = 1) with the youngest participant of 26 years (*n* = 1). According to the status of the family relationship with the person they were caring for, the majority of the participants were daughters (*n* = 8) providing care for their mothers (*n* = 7), with one woman providing care for her father. One participant provided care for her husband and one provided care for her grandmother. Thirty percent were caring for a person living with vascular dementia (*n* = 3), and 30% for Alzheimer's disease (*n* = 3). One participant was caring for a relative living with vascular dementia which had not been identified, but all signs were consistent with frontotemporal dementia; vascular dementia with Alzheimer's disease (*n* = 1); dementia with late-onset Alzheimer's (*n* = 1) and an unspecified form of dementia, including the heart failure, diabetes, optic nerve damage, and hypertension (*n* = 1). The majority of participants were living together with a person who has dementia (*n* = 6, with one participant commenting: “*Only leaving when I go to work*”), and 40% lived separately (*n* = 4). Among the carers who were living separately from the person they cared for, the regularity of their visits, as stated by the participants, was as follows: “*4 times per week”* (*n* = 1); “O*nce every two weeks, on weekends. It's a minute of respite for dad. And if he is going to the sanatorium, I'll take some vacation and take care of her*” (*n* = 1); “*2 times a week and during the weekends spending time with mom for 2 or 3 days”* (*n* = 1); “*Very varied: always on weekends and sometimes once or twice during the week. In another city. …*” (*n* = 1).

Majority of participants were caring for a person living with dementia for 1–5 years (*n* = 8; among whom seven care partners indicated the exact duration, with an average of 4 years), and two participants had been caring for 5–10 years. Participants who reported caring for a person living with dementia for 1–5 years provided statements such as: “S*ince 2016*”; “*3 y*.”; “*1.5 y*.”; “*5 y*.”; “*About 3 years. I didn't understand it before, that stealing, knocking, etc*.”; “*A couple of years*”; “*Soon it will be 5 years until moving her to my home. She forgets to eat, does not take care of herself. She lived in the district area. Immediately she was considered for a nursing needs, a special needs group II. Decided that it was better to take her to my home. Before from May to July I have lived with her at her place*.”

Half of the participants were working besides caring for a person living with dementia (*n* = 5), and five participants were not. Participants stated: “*I leave for 3–4 hours. I can go out, very limited*”; “*… I have left my work due to need to take care of my mother... I took 2 kittens (there were 3) and 1 puppy to live with us as they were her pets*”; “… *Mom can sometimes panic when I leave. Great anxiety. While I am at an event, thoughts can constantly revolve around mom—what she is doing, is everything okay. Great stress…*.” “*I work 4 hours a day, I hire a woman who comes. The job is music. If it weren't music, it would be very difficult…*,” said the carer who moved to live with her mother in order to take care for her. Thus, this study focused on a care partners of people living with dementia, who are daily exposed to a variety of care and well-being challenges (32, 33).

The majority of participants refused to accept the financial support to cover transportation costs (*n* = 7), but requested funding for a care assistant at home (*n* = 6) to be able to attend the on-site meetings. None of the participants had taken part in photography art activity before (*n* = 10) and no one in a group had previous Photovoice practice experience (*n* = 10) (Table 1).

**Table 1:** General characteristics of participant group.

Characteristics	Participant group *n* (%)
Gender	
Female	10 (100)
Place of residence	
Large city	6 (60)
Medium city	1 (10)
A small city in a remote area	1 (10)
A village (in the outskirts of a large city)	1 (10)
A small city (in the outskirts of a large city)	1 (10)
Age groups (years)	
25–39	1 (10)
40–54	5 (50)
55–70	4 (40)
Family relationship to the person living with dementia	
Daughter	8 (80)
Wife	1 (10)
Granddaughter	1 (10)
The form of dementia the person they are caring for has	
Vascular dementia	3 (30)
Alzheimer's disease	3 (30)
Vascular dementia has not been identified, but all signs are consistent with frontotemporal dementia	1 (10)
Mixed dementia (Vascular dementia with Alzheimer's disease)	1 (10)
Late-onset Alzheimer's disease	1 (10)
Unspecified dementia with comorbidities: heart failure, diabetes, optic nerve damage, and hypertension.	1 (10)
If a care partner lives with the person who has dementia	
Yes, I live together	6 (60)
No, I live separately	4 (40)
For how long have family care partners been taking care for a	
person living with dementia (in years)?	
1–5 years	8 (80)
5–10 years	2 (20)
Work status (if also employed while caring for a person living	
with dementia)	
Yes	5 (50)
No	5 (50)
Previous experience with photography	
No	10 (100)
Previous experience with Photovoice	
No	10 (100)
Are you willing to receive financial support to cover	
transportation costs for attending on-site	
Photovoice activities?	
Yes	3 (30)
No	7 (70)
Are you willing to receive financial support to cover the costs	
of a care assistant at home to attend on-site study	
Photovoice activities?	
Yes	6 (60)
No	4 (40)

Participants commented on their experience of taking photographs: “*Just to take a walk and to take pictures on the way*” (*n* = 1); “*Once in a while, by the feeling, by the prism of what is beautiful. I like what is beautiful, with a thought. I try to find an angle, lighting. I'm not always taking pictures, but sometimes*” (*n* = 1); “W*ork with people with visual impairment—blindness, in trips*” (*n* = 1); “*Previously more, but now less. From time to time we capture our mother for documentation. I document. It's not something uncommon to me. It is not shared elsewhere more. Those who face this disease, their relatives face the other side of it. Others ask how your parents are doing, out of politeness, but no one wants to hear the answer. When they see it in reality, they often say that ‘I had no idea it was like this.' We don't tell them because no one will understand. So that people would start think about it. When I hear someone saying ‘I forgot, maybe I have Alzheimer's,' I want to go closer and shake them. There is no worse disease for me. A person receives through a visual, not through a text. Whether on a phone or a camera*” (*n* = 1); “*I take pictures for my own pleasure of sunset, forest, pond. It melts into beautiful balls*” (*n* = 1); “*As a necessity, but not constantly photograph. I have a love of life who takes photos. Children take photos. I look at those photos, they are beautiful. And I thought, so do mine*” (*n* = 1); “*My camera is of an old phone. I take pictures at work, I do a very good podcast. Taking pictures of stuff. Looking for an angle*” (*n* = 1); “*I try to take pictures—more artistic. Since long time ago, but artistic*” (*n* = 1); “*Instantaneous*” (*n* = 1).

In addition, participants' answers to the question, “What interested you in this project and made you decide to participate?” varied, including a wish to learn new things, engage with the arts, and gain or exchange experiences: “*Experience*”; “*I want to share my experience, that even in the darkest night you can find a ray of light*”; “*Interested in these possibilities to participate in a creative process, to tell a story through photography, in a safe space, using metaphor, figurative language to explore essential issues related to the care of a person with dementia, the caregiver experience in care*”; “*To share experiences while caring for a loved one. To get useful advice for myself. I am interested in photography*”; “*The opportunity to communicate with others and improve your photography skills*”; “*The opportunity to look at dementia from a different angle*”; “… S*haring experiences while caring for their loved ones, trying new experiences*”; “*I think that photography can be a very good way of self-expression for someone who does not have time to develop other creative abilities. I have been interested in this field for a very long time, but I never felt like I had the right opportunity to try to learn it, it would take enough time and really delve into it. I did not dare to try, and this would be a great opportunity*.” Focus on care partners: “*Attention to caregivers*.” Expressing societal stigma: “*The topic itself. Because in reality, people really don't understand what people who care for people with dementia face*.” Desire to be with like-minded people “*Community …*.”

### 3.2 Identified themes

Our research findings, presented in this article, revealed that, in conversations to discuss the photos, a number of themes referencing the supporting aspects to the well-being of care partners of people living with dementia in a care relationship emerged. Below are the core themes.

#### 3.2.1 Reconciliation and acceptance

A number of participants used photography to express feelings of reconciliation and acceptance (Figures 1–13).

**Figure 1:**
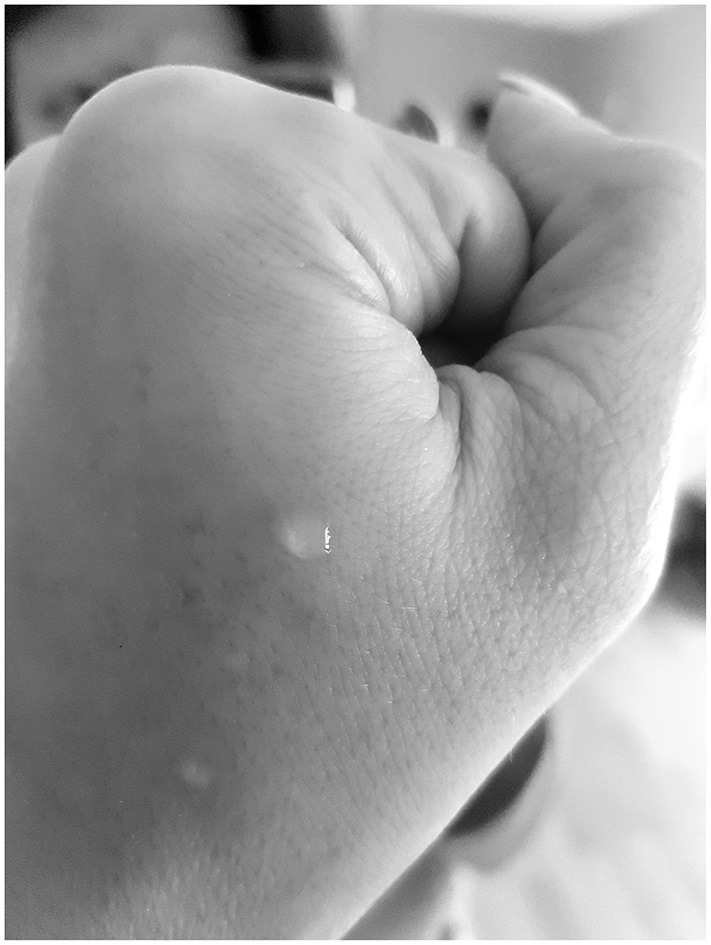
Jovita, “*Anger”* *You search the whole house for hidden objects, collect food leftovers from the bed, take toilet fresheners out of the fridge, persuade her to eat, to wash, and maybe for the 10th time you answer the same question again… I lose control… I get angry… But how could you get angry? Then guilt creeps in. She's your mother! You're healthy, and she is the one with the illness. I understand that it's the disease, but sometimes it feels like she's doing it on purpose… Then comes anger at myself. How could you? You cry. The greater the anger, the deeper the self-blame. I tell myself: I really won't get angry again, I really won't scold her. But then it's the 10th time… You can never predict when and what will happen again*.

**Figure 2:**
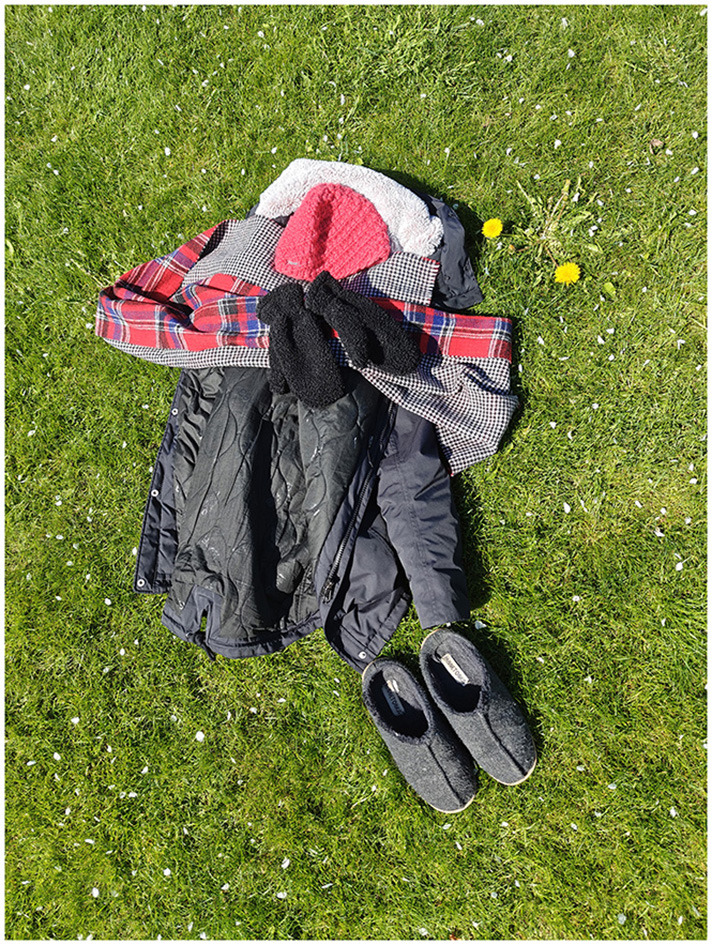
Vaida, “*Winter in Summer. Summer in Winter”* “*I will probably die,” she often says*. *As a little girl I saw my mom drinking coffee in the kitchen, the radio playing, and she is cheerful, carefree, always in a good mood*. *Mum always looked polished, with her makeup done, heels on, and dressed up*. *Friends used to say: “Your mom is so nice!” Out of all my friends, I had the best mom. The person she once was is no longer here*. Now, whether it's cold or hot—she cannot understand how everything is anymore… *A feeling of hopelessness*. *People stand and stare*. What might they think? *The scariest part is that others have no idea this is an illness*. *They might laugh, they might insult*, *God forbid they might hurt her*. *I am afraid that mum could get hurt*. *It's hard to accept*. *I try to block it out, not to think, not to analyze*. *It is what it is*. *I wish it weren't so*. *When I learn to accept, then I won't pay attention anymore*.

**Figure 3:**
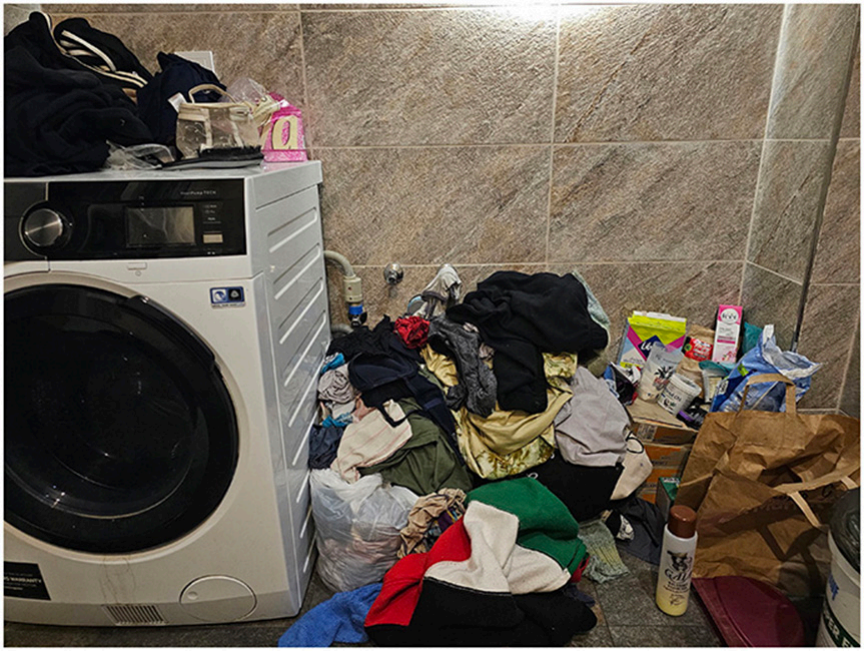
Aušrinė, “*Sisyphus Is Still Happy”* *The bathroom is a manifestation of what can never be finished. It's still without cabinets, without anything… without a foundation. The lack of a foundation allows disorder to accumulate. There is noise in my head. Physical, inner noise. A lack of system*. *I feel that everything is my responsibility*. *Every day, you have to do the same thing over and over*. It's strange to encompass so much… *Each day is a simple, domestic tragedy*. *Work and family. You are in a small circle that isolates you. You are what you return to every day: a limited number of people, your earned money, your work. Caregiving becomes an internal, hidden, isolating process*. *People lack the energy and time to be human*. *Sisyphus is happy because it's not worse than it is. He has already accepted the absurdity of his situation and can roll the stone with satisfaction. The only way to survive is to live with a positive attitude*. *I chose not to be sad and to move forward*.

**Figure 4:**
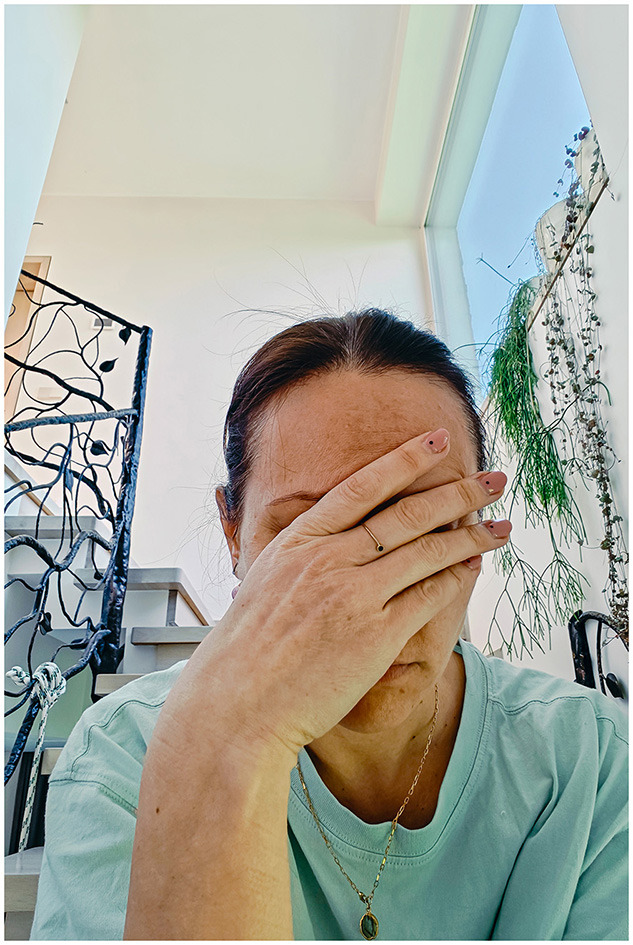
Vaida, “*Why Me? Why My Mother?”* *It seems like everything is fine, but it's not*. *When you don't know what to say or what to do*. *It's sad that it happened this way*. *It's sad that this happened to my mother*. *Sometimes I feel sorry for myself. But I feel most sorry for my mother*. She is 65, could be enjoying life, living fully. Now she finds joy only when she feels safe and calm. She is happy when I give her something to eat… *She wants to go somewhere. But I can't take her… Inside—a tornado. She grabs and is swept away, can't escape*. … *It irritates you, but you can't change anything*. *Time heals, working on yourself helps*. *I am patient—as much as one can still be*.

**Figure 5:**
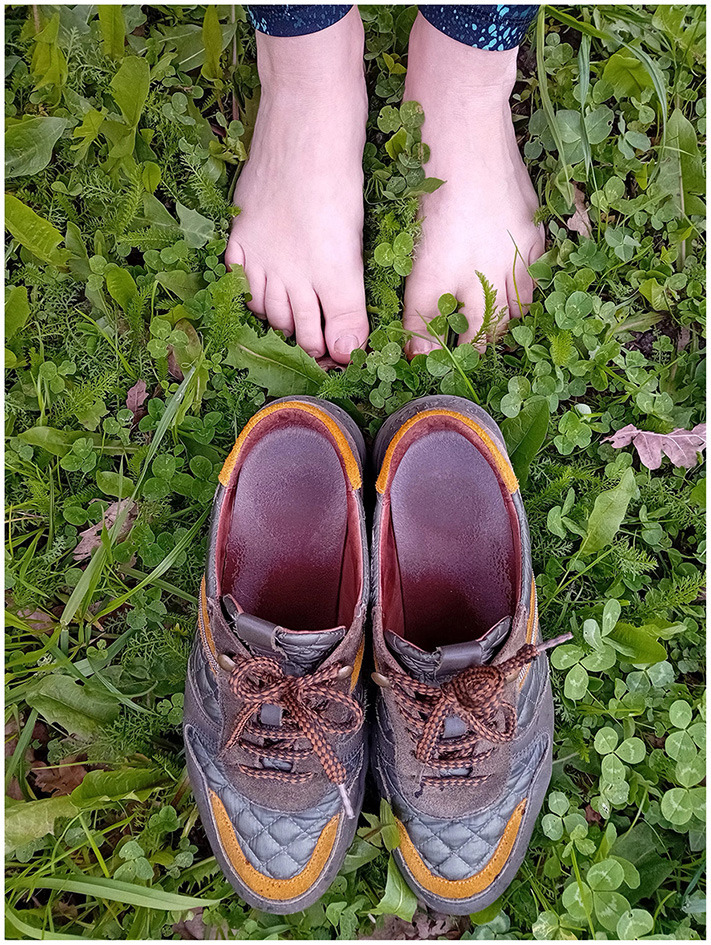
Ingrida, “*A Way to Return to Yourself”* *Nature accepts you as you are*. *It helps you feel the ground beneath your feet, to collect yourself together so you can move forward: to go back to work, to take care of your family*. When I first learned about the illness, there was a stage of mourning. Now—the caregiving stage, or “rollercoaster.” There were bad days and good days. Now—unpredictability. Good minutes and bad minutes. Constantly stepping out of the comfort zone… *It is still hard to accept that this is my life*. What could I have done differently so this wouldn't have happened to me?

**Figure 6:**
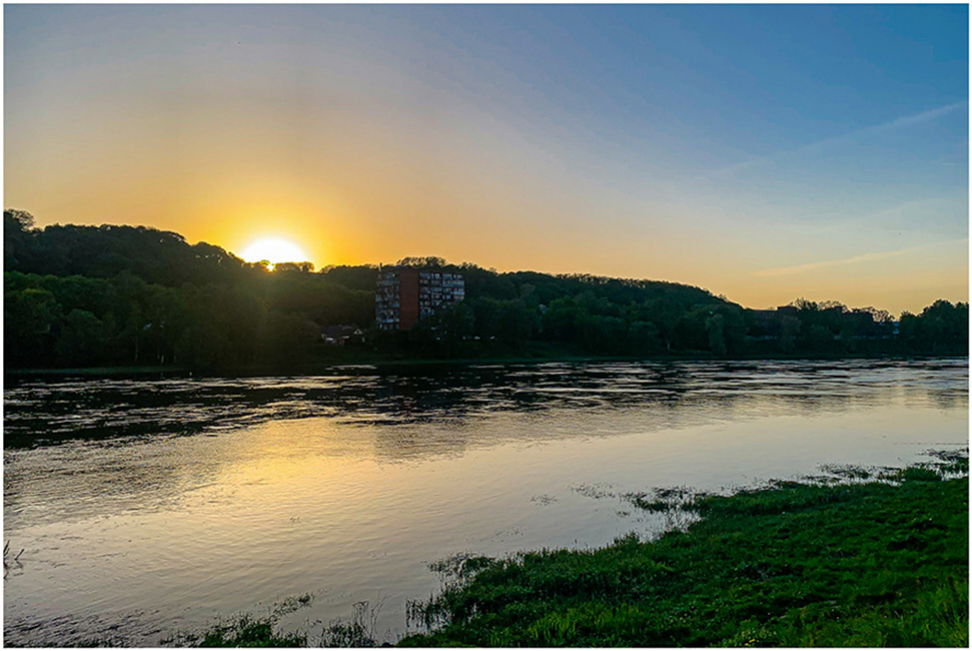
Žaneta, “*Everything Has an Ending… Even a Day”* *Everything comes to an end… This isn't about death*. *Everything will end eventually*. *I have to live through the present moment*. *Every task has its ending, every moment, every period*. *I cherish each day: that my mother is here, that she smiles when everything is going well*.

**Figure 7:**
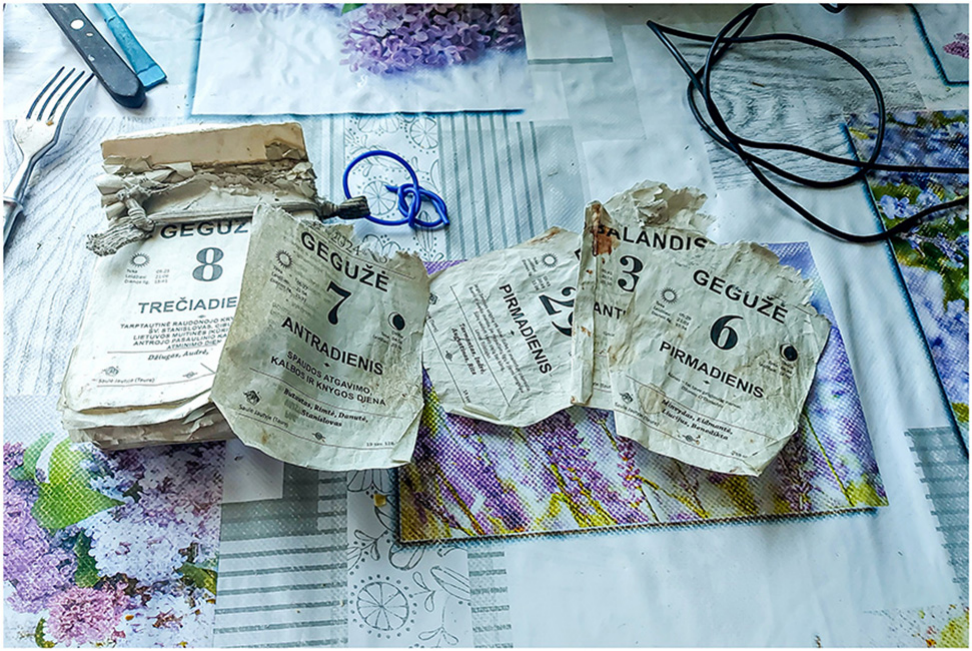
Vitalija, “*Time—the Most Important Thing” (1)* *As soon as you step through the door, the first thing she asks is to organize the calendar*. “*Why do you tear off so many pages?” I ask*. “*I don't know.”* I ask her to help track the days, to help me arrange it… *Together, we organize and stick the pages*. “*In the evening, when you go to bed.”* She sleeps a little, then gets up and asks, “Is it evening already?” “*No,” I say, “it's still morning.” We try to figure it out…* *She asks what day it is. And this happens a hundred times. I tell her the day, and she calms down*. *You have to accept the person as they are, not try to change their thinking*. It took me more than a year to accept this… Earlier, I didn't listen and wanted things my way: you need to wash, you need to tidy up, you need to be here and there. I have to accept her questioning, that she will keep asking, that we will go back to the calendar… *If you don't answer what is most relevant to her, then it's bad*.

**Figure 7:**
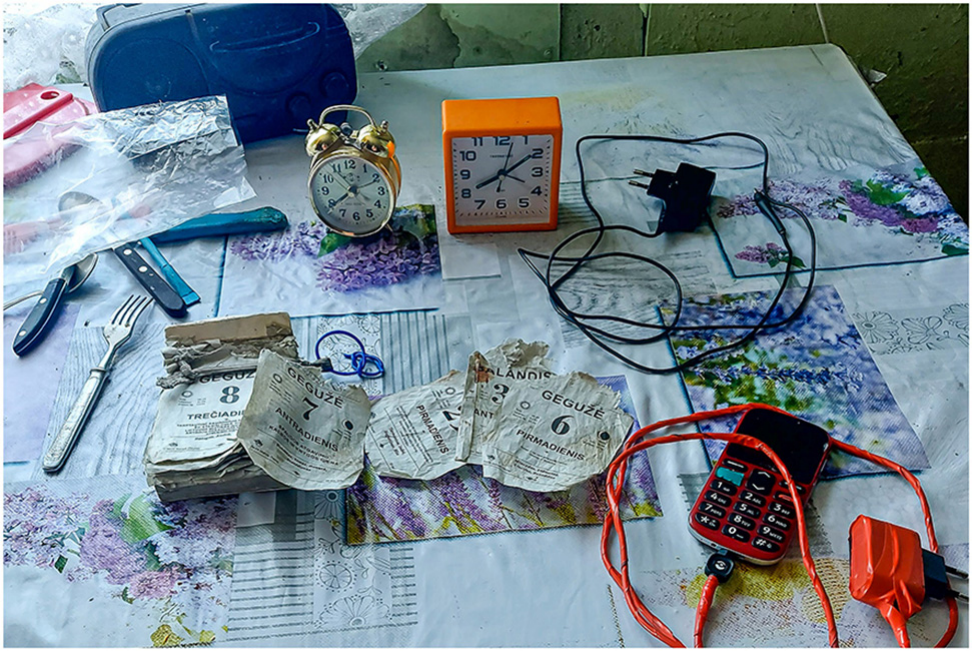
Vitalija, “*Time—the Most Important Thing” (2)* *At first, two clocks were enough*. *Then we bought maybe five more, because something was always wrong: if it was even a minute off, it wouldn't work for her, and she would start twisting it*. *When she turns it, she no longer knows the time. I bought one that can be wound up. She learned how to do it. And she also gets to move her fingers*. *She needs two chargers, two phones, so she can make calls*. *The phone cords were identical, so I wrapped one in tape: red phone—red cord*. *She uses the phone and charges it. If something goes wrong, I call a neighbor—she goes to check that everything is okay*.

**Figure 7:**
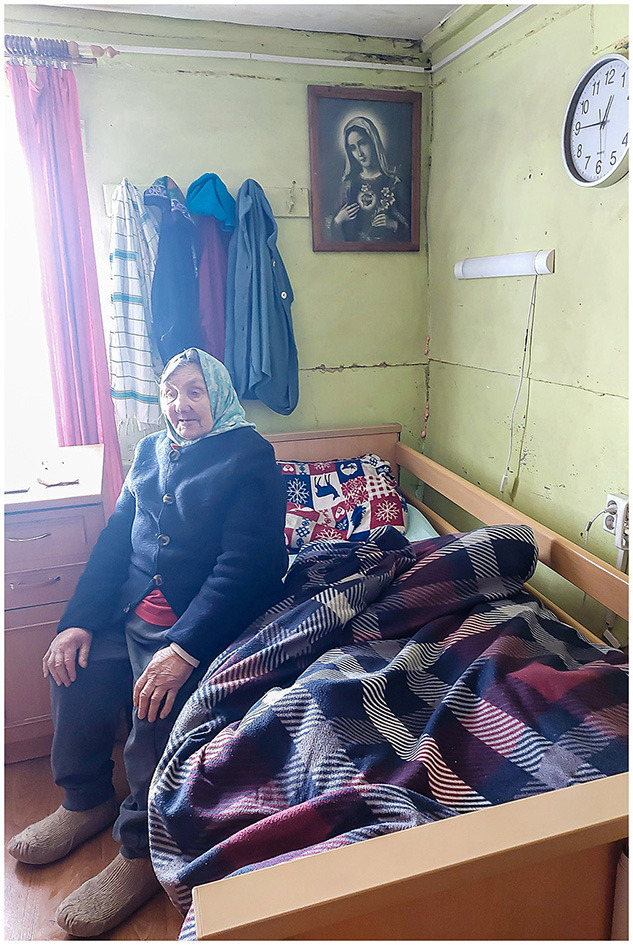
Vitalija, “*Time—the Most Important Thing” (3)* *Her bed, her room—the safest place. She feels best in her own bed… She sits. Feels safe*. *We hung a clock on the wall by her bed*. *She can't turn it*. When she asks the time, I say, “Look, there's a clock on the wall by your bed.” *The clock has large numbers*. She goes, looks: “Five to seven…” Soon after, she asks again: “What time is it now?” *By where she sleeps, on the side—there's a spotlight*. If you offer something new: no, don't need it—she got very upset, when I brought it: “Take it, get it out of here.” *Now the light shines, and she can see what time it is*. Mom always watched when the sun rises and sets. “The sun sets at 9, go home.” *At first, I thought she wanted to send me away, but now I understand—it's her care: the child leaves, so that you are safe*.

**Figure 8:**
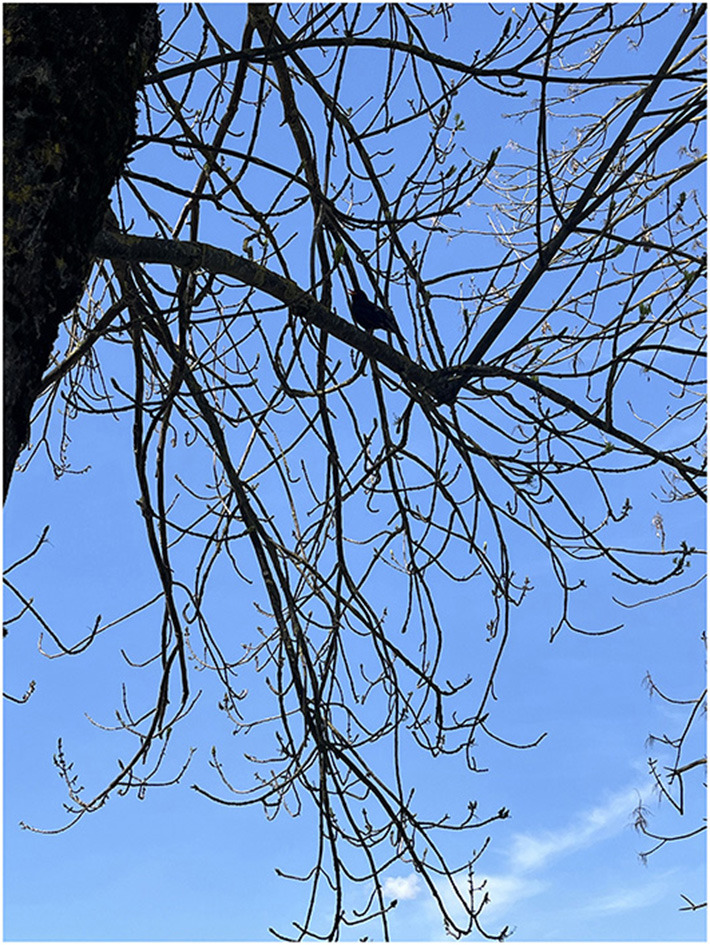
Janina, “*Tangles”* *A tangle of illness, institutions, trips, unfinished tasks. It is what it is. What I can no longer do, I no longer need to. I had plans to grow old gracefully, to sail on the Nile. I thought, like everyone else: I'll retire, I'll travel, I'll go, I'll sail… You plan one way, life takes another*. I am like that little bird without a nest. Now there is only one branch. Not much to bring along, only I decide… Everyone can listen, but you decide what to do yourself. Many say: “Don't worry—he's sick.” If I just want to vent, I hear again: “Don't worry.” *If you haven't experienced it, haven't lived alongside—you won't understand. No one will understand, whether from a joyful story or a sad one*. *Which branch—thinner or thicker—should I squeeze through? There is a choice of which branch to sing on—there are equal, thin, bending ones. I still have a branch*.

**Figure 9:**
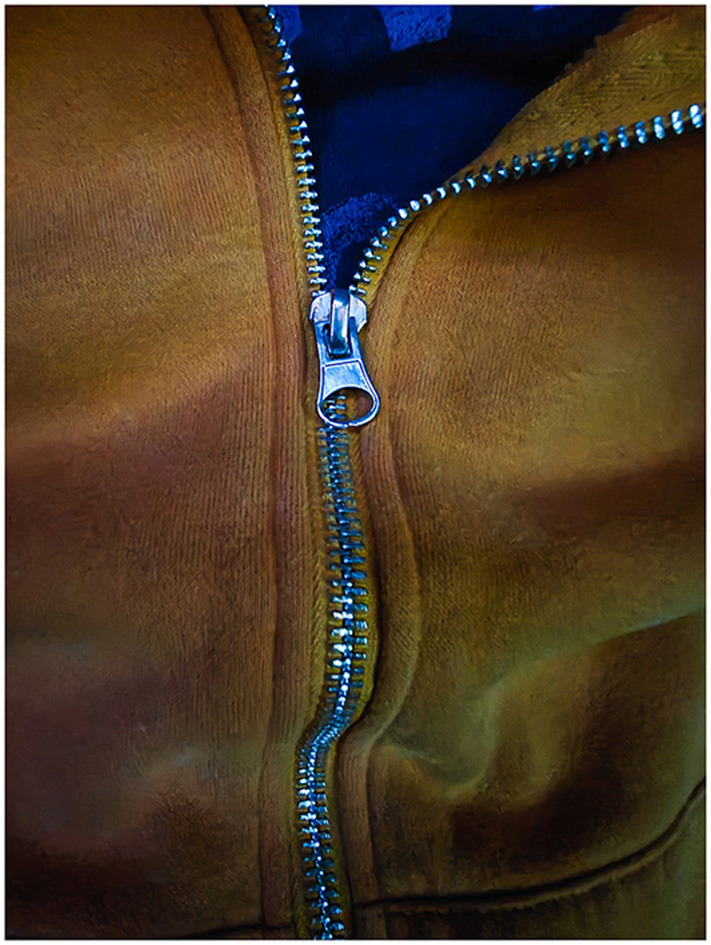
Vaida, “*Pulling in Emotions”* *I have to block my thoughts, not analyze, not dig*. *Hide thoughts and emotions*. *Suppress memories of what she used to be, so it won't be hard*. *Hide all of this from myself and others*. *I must not talk, complain, or tell*. *Those who haven't experienced it won't understand*. *I “withdraw” in front of those I can. The only ones I can talk to are my brother and his wife*. *And the children! They are wonderful. With them I have the same kind of bond I had with my mother*.

**Figure 10:**
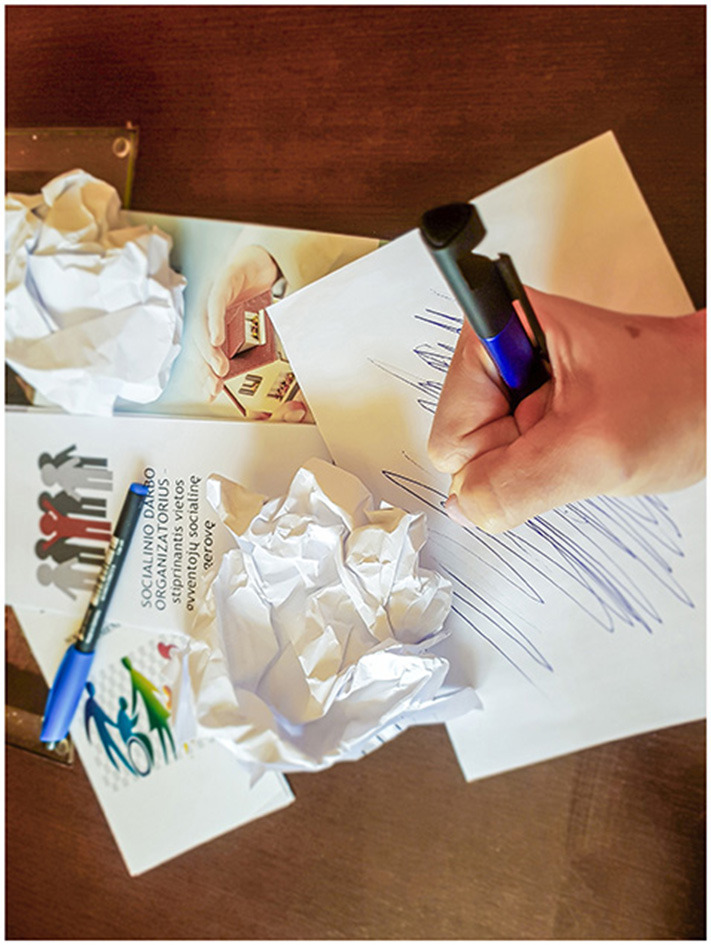
Jovita, “*The Struggle”* *Anger. Banging your head against the wall. Fighting for your rights*. I applied to the municipality for services. I was told, “We're very sorry. But there's no funding, no services.” *They didn't even accept my application. By chance, I found out that the municipality is obliged to accept applications. How convenient: no application—no need*. *I send a written application and receive a reply: “Your application has been accepted, but as I mentioned, there are no funds.” First achievement: the application was accepted*. *I organize home help myself, persuading a private company to start providing these services in my mother's town*. *It turns out there is a need for such services; after me, they started providing them to others too. There is now some support from the municipality as well—they cover half the cost of the services*. *Caregivers don't always know their rights*. *Many lack awareness and competence*. “*I found out for the first time that there is such a disease,” said an employee who came to provide services for the first time*.

**Figure 11:**
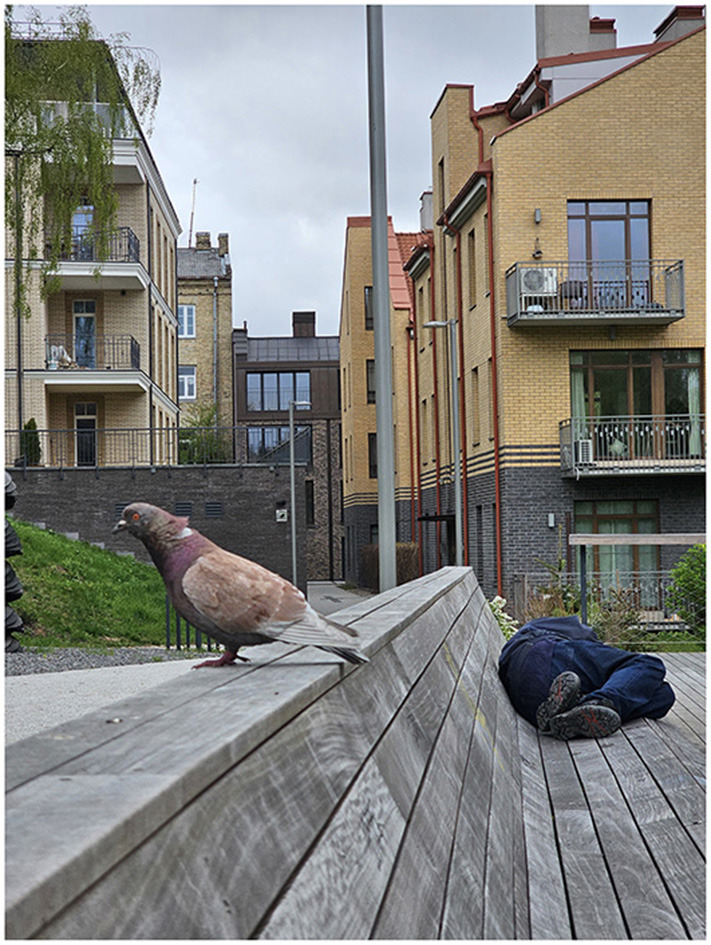
Aušrinė, “*The Abandoned”* *In the past, pigeons were domestic animals—cared for and loved*. *When there they were no longer needed, we abandoned them. Members of society often look down on those they consider useless*. *A society is only as strong as its most vulnerable members*. *Disability, illness, loss of function, and so on are part of life*.

**Figure 12:**
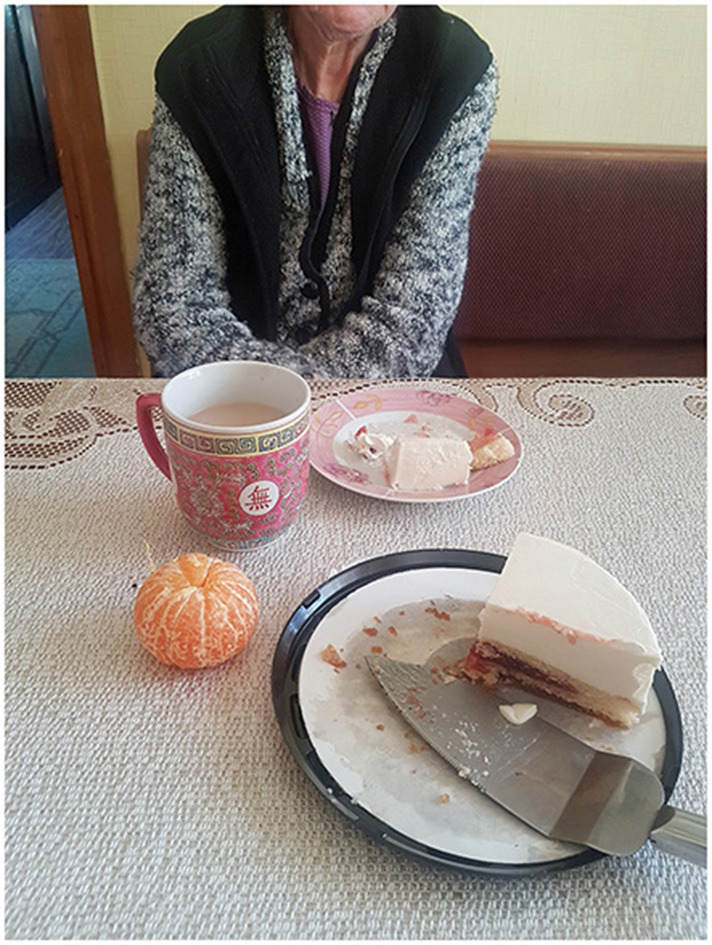
Jovita, “*A Sad Birthday”* During holidays, she used to set the table, and her friends would come. Now her friends call me and all they talk about is the illness. They say, “Hang in there, if anything—you have my number.” And it sounds like, “Invite me to the funeral.” I'm tired of pity. I would like to say: “Come, spend time together while we still can! You can't socialize over the phone, and you can't celebrate a birthday that way.”

**Figure 13:**
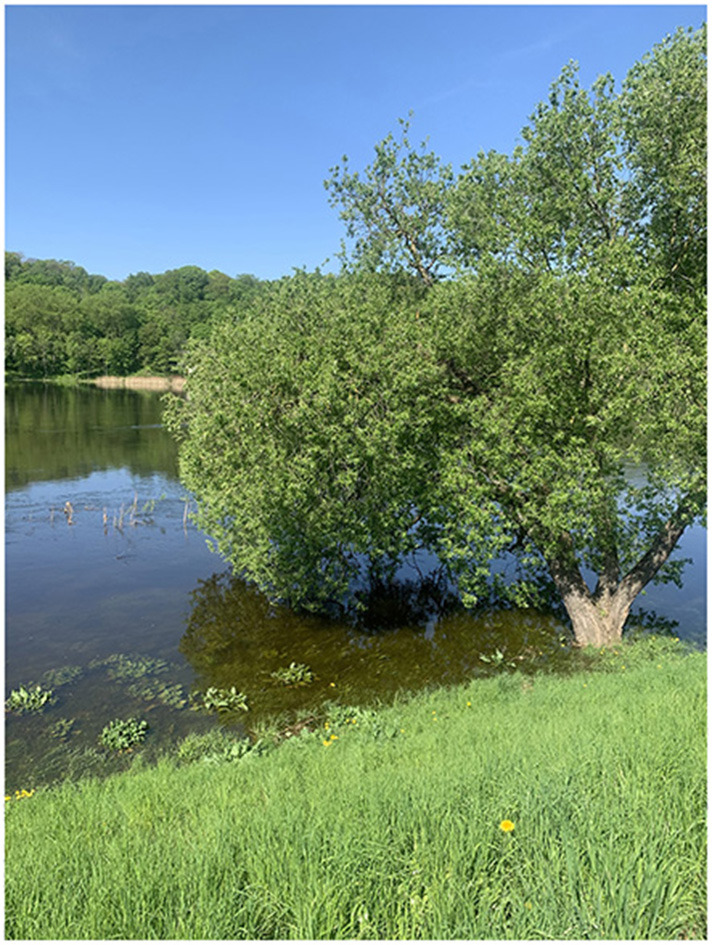
Žaneta, “*Old Age as a Tree: Bent by Burden, Beauty Unchanged”* *My mother is aging, but to me her beauty doesn't change. She can no longer walk, but she is still beautiful to me. The older generation of mothers never thought about preparing for life in a nursing home. They never thought about how not to burden their children—the idea was, we will live together and care for one another*. Maybe now it's considered “beneath you” to care for your parents? Doctors and acquaintances have told me: “Why do you exhaust yourself so much? You can hand her over—then just come and visit.” *Those who provide care at home do need rest. But can I place her in a medical facility and feel at peace? Right now, I cannot. I don't have the trust that everything will be fine: that her diapers will be changed as often as needed, that she will be offered water (since she doesn't ask when she's thirsty—sometimes she drinks, sometimes she doesn't, so you have to offer often), and so on*. *I fear that my mother might be harmed… I see how many people working in the care system have become numb to the fact that this is a life—a life worthy of respect. Compassion is lacking*. *And you can't fight the system*. *Now my mother's teeth hurt. Normally, when you're in pain, you can go without waiting… But when an old woman arrives, unable to walk, they take a look and say: “There's no inflammation,” and send her away. But she's in pain. My mother is in pain, she doesn't eat. My heart breaks from that pain, because I feel it all*. *No one cares. If it's not your mother—no one cares*.

#### 3.2.2 The aspiration to maintain one's inner freedom and well-being

Another major theme identified in the study was the aspiration to maintain one's inner freedom and well-being (Figures 14–22).

**Figure 14:**
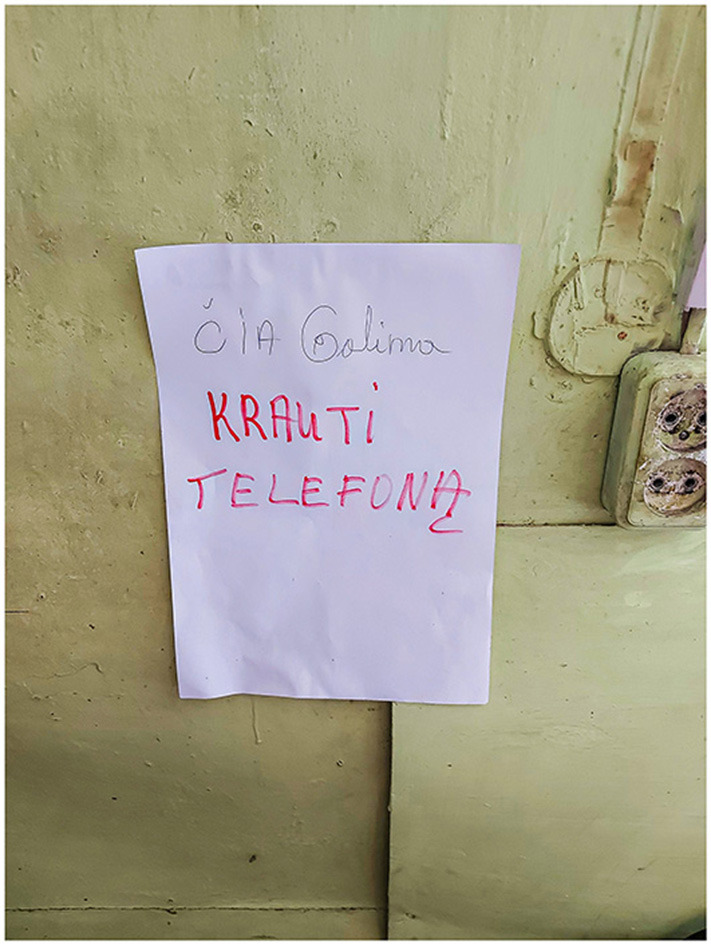
Vitalija, “*When she doesn't know, she becomes restless. Clarity comes when she reads and sees.”* *Before, I used to call the neighbor more often: she didn't know how to charge the phone or do other things*. The psychologist advised: “Let her do it herself… so she understands. Don't do things for her.” *Now I feel calmer. I need to call the neighbor less, because she manages to do many things on her own*. *The neighbor, seeing how I manage, said: “I did myself a disservice. Taking care of my mother, I wanted to hand everything to her*. Mum was bedridden, didn't walk at all. She never moved on her own…”

**Figure 15:**
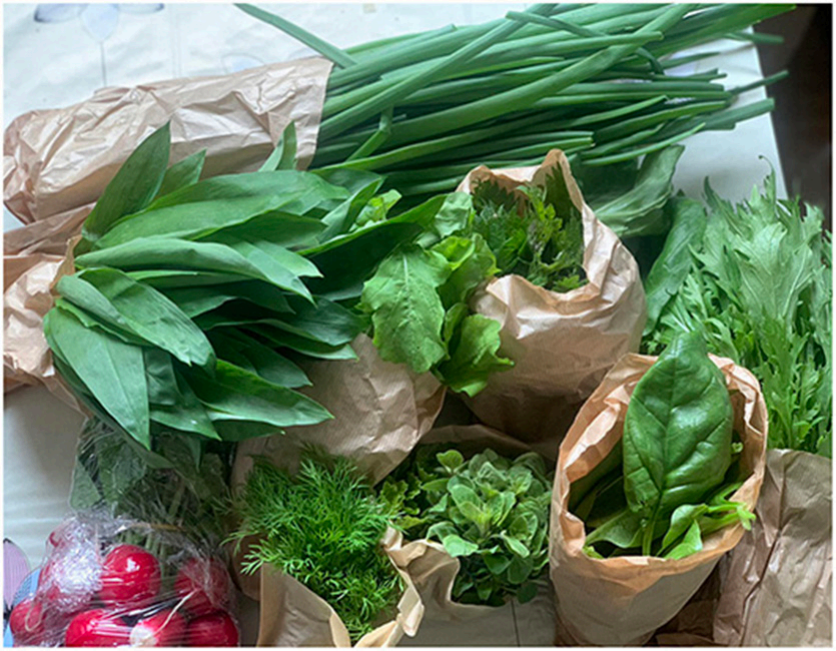
Žaneta, “*How to Recharge Joy for Life”* *The greenery—so that I can be stronger. This is my daily joy. It's a piece of strength that I receive from nature*. *Those batteries run out… Who will recharge them? The load at work and here is similar. But here it's my problem*. My relatives say: “How hard it must be for you, how hard it must be.” *It's not really hard, not like that*. Sometimes it's hard—you rest and then go on… *It's very hard when I feel unwell*. *When I feel healthy, strong, then it isn't hard for me*. Yesterday I spoke with a relative: “How are you?” I said: “Good.” “*How can it be good? It can't be good…”* It is hard. Really hard. But who cares?

**Figure 16:**
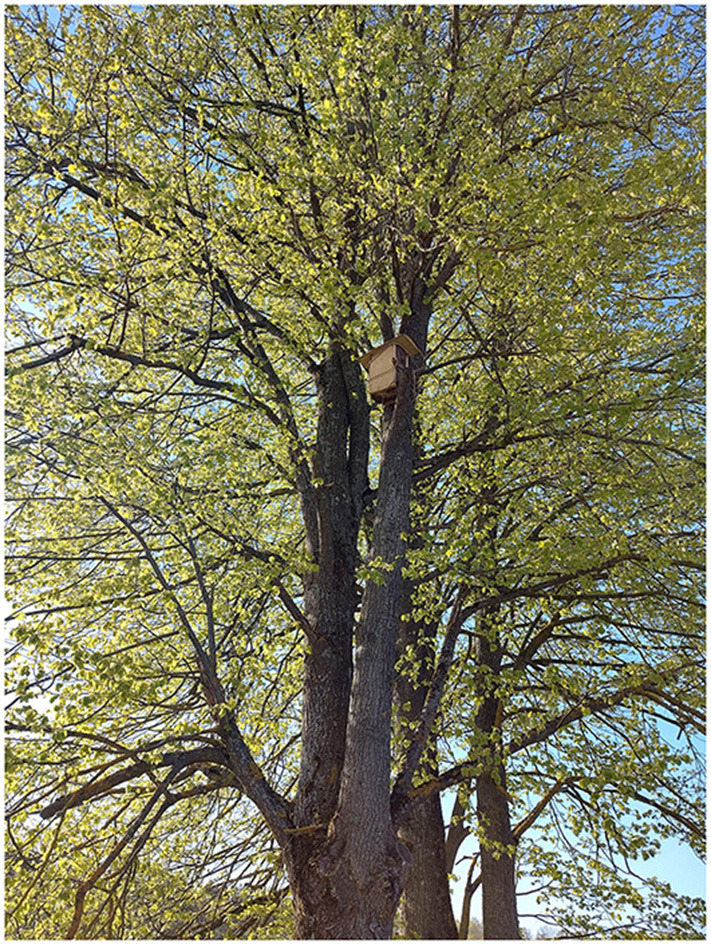
Vitalija, “*Just to Soothe Myself”* *The thick lindens planted by my grandfather protect from the sun, you can hide when it's hot*. *I wanted those birdhouses so I could watch how the little birds settle in*. *I waited for the starling to make its home*. *Now I watch how it flies, how it feeds*. *It is relaxation, when you feel you can't go on anymore*.

**Figure 17:**
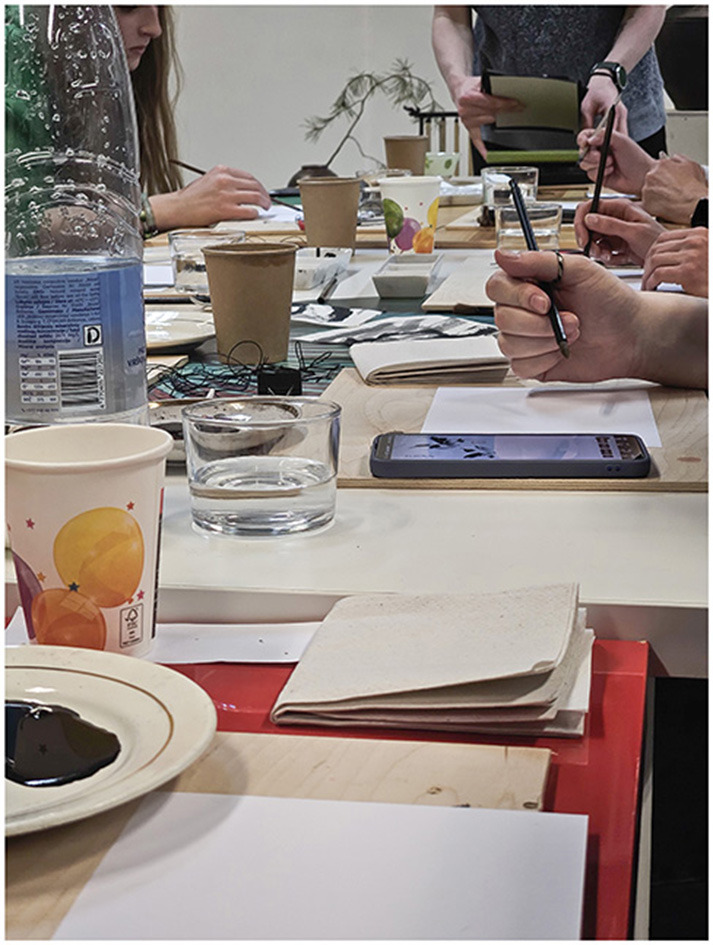
Aušrinė, “*Forced Slowing Down”* *After a long time, I left home to meet a person I hadn't seen in a while*. *It was very difficult to draw with ink. The more tense you feel, the harder it becomes. After a while, you realize that you have no choice—you must relax, otherwise you can't control the brush*. *Life is tense, fast. I have to live for two people, feeling responsibility for myself and for another. But it's impossible to rest for two people*. *Rest is one step forward. Returning to everyday life is two steps back*. *Who could help change this situation? A fairer division of work, respect for responsibilities, and empathy. In the family, there is a lack of understanding and recognition of this syndrome. What is dementia? How does it manifest? Until that exists, I continue to worry about how the person I care for is being looked after when I'm not around*. *Artistic activity is also important for the person being cared for*. *My dilemma: can I make time for it? Other people perhaps cannot allow themselves that at all*.

**Figure 18:**
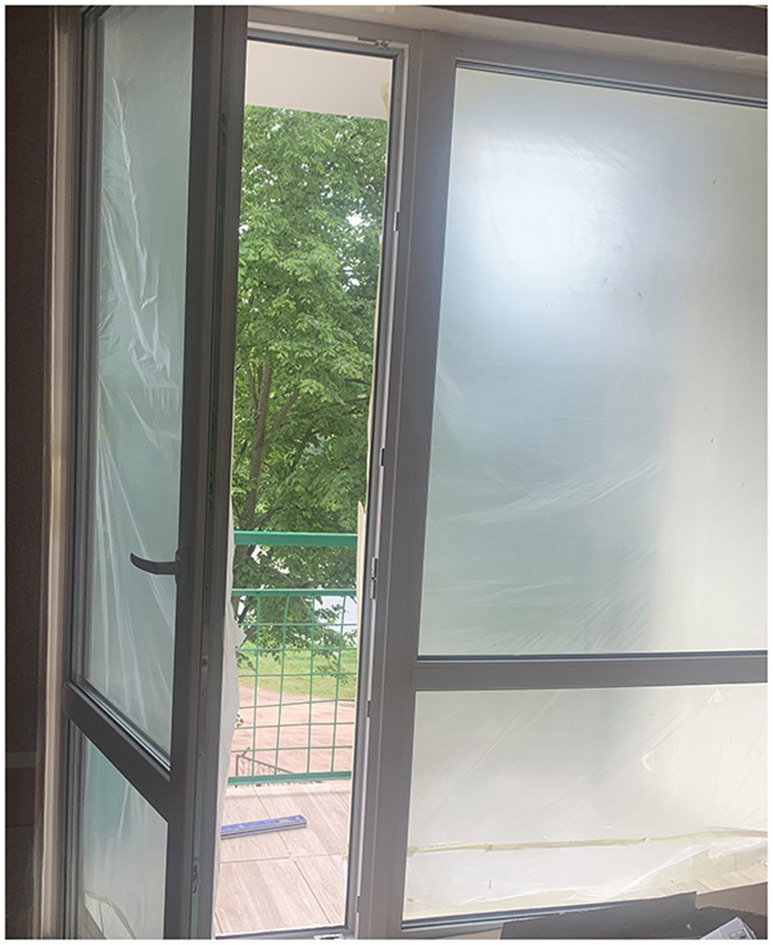
Žaneta, “*Longing for Light…”* The handyman came. He asked: “What do you want me to fix?” “*The balcony!”* *These houses belong to my mother. The balcony had been glazed to protect us from the sun and the cold. That's why it felt gloomy, dark*. A feeling of being trapped. Along with it—loneliness, routine, anxiety… *I wanted to let light in—life goes on. When that feeling came to me, everything just exploded*. *I couldn't find a way to have access to nature, freedom, fresh air, light*. *You have to create it yourself, make it happen so that there is light*. *When the balcony was opened, I was so happy. I had never felt that way in my life. I feel stronger because of what I did*. “*Why do you need that balcony?” someone asked*. *And I just suddenly went ahead and did it. And I felt so good that I just did it*.

**Figure 19:**
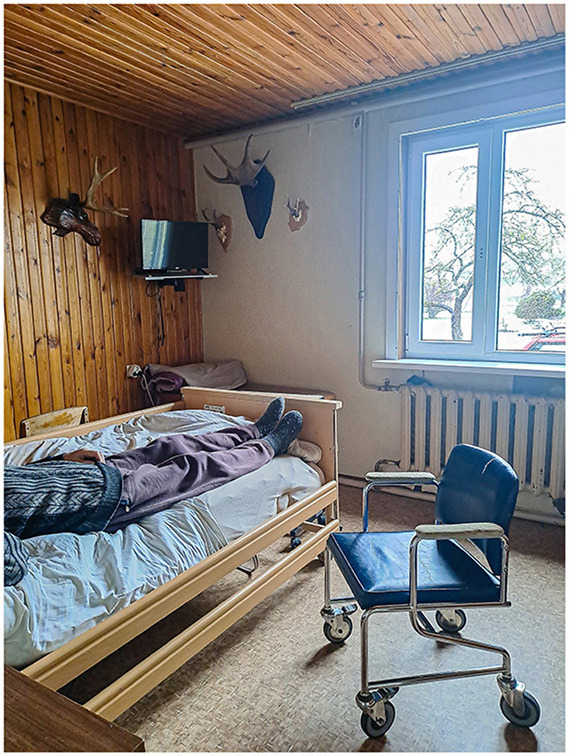
Rima, “*Rest”* *Lying down to rest legs after moving around, after walking through the house*. When he rests, I try to go outside or sit in the fresh air…

**Figure 20:**
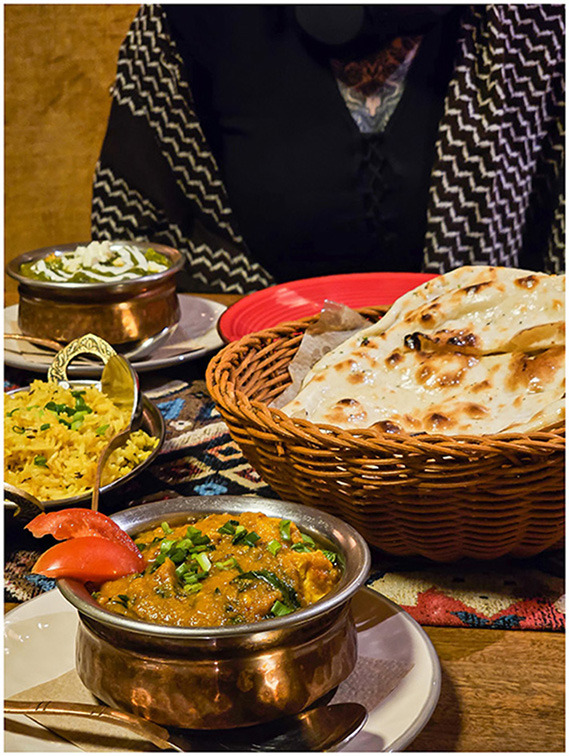
Aušrinė, “*Bitter-sweet”* *My sister and I. An emotional evening. Food. Time together*. *One of those days when we allowed ourselves to go out. Not for chores, just for the sake of it*. *It was a choice*. We do most of the caregiving, and if we're not home, we get very anxious… *This time we decided to treat ourselves to a good meal. It was incredibly delicious. It's great when someone else makes your food and you don't have to worry about cooking*. *I prepare food for my sister and grandmother*. *My father cooks in the evenings*. *At home, we don't eat together at the same table*.

**Figure 21:**
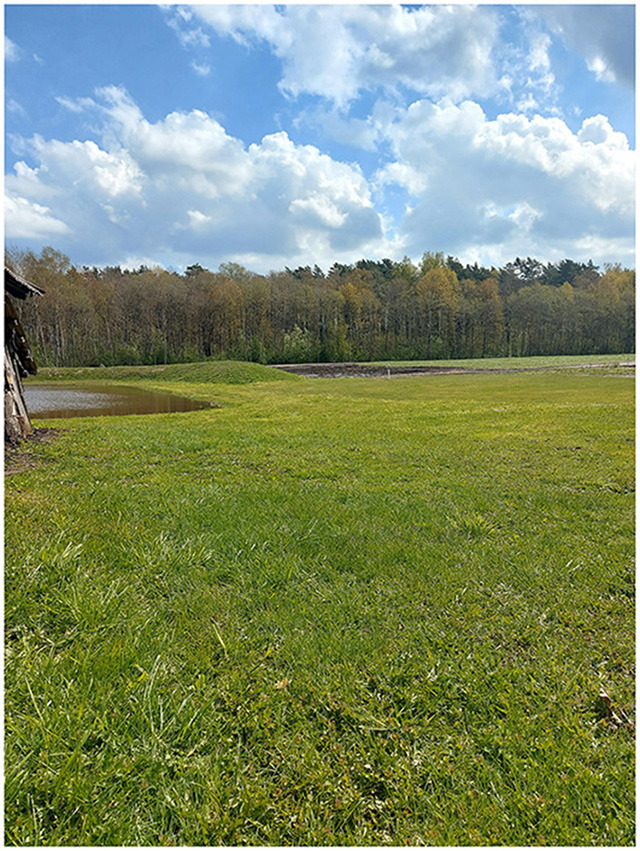
Vitalija, “*Nature, Where You Recover”* *This is my place of calm*. *When I start getting irritated, when I'm overtired*. I've already said it, I've already written it, but the questions don't stop… *When I see I can't hold myself back anymore, I go out to the little pond and walk around it about six times*. *Now it happens less often… before, I was always unrested*. *I wanted to do too many things*. *I reduced my workload*. *I try not to come when I'm overtired, so I won't be angry*.

**Figure 22:**
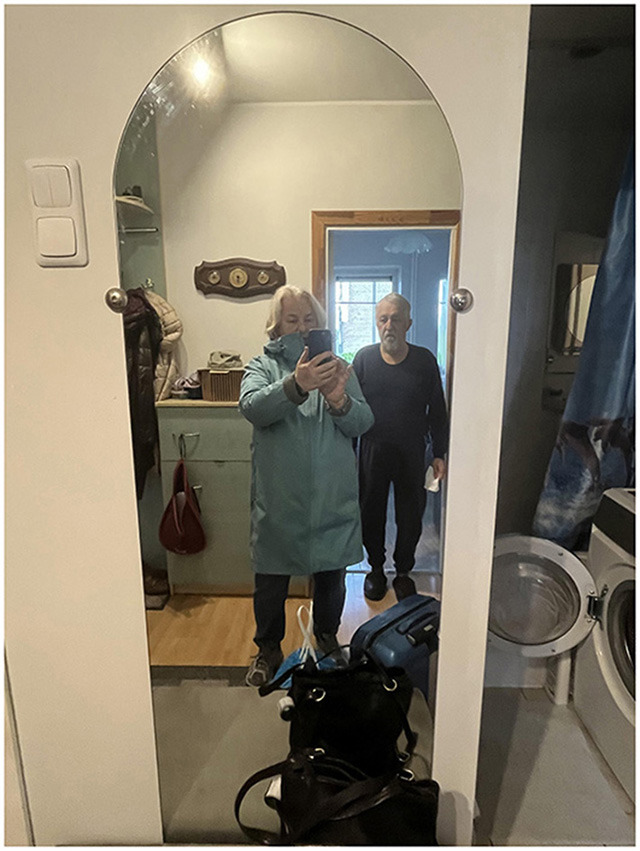
Janina, “*Maybe This Is the Last Time I Can Leave Him for a Week…”* *Every year I go to a sanatorium twice, to recharge myself. For a week, no longer*. *Before the trip, I have to start announcing the day I'll leave, at least a few weeks in advance. And I say it every day*. *When only a few days are left, I start preparing… I begin explaining that he'll have to get his own breakfast that my granddaughter will bring warm lunch. I ask that he doesn't leave the key in the lock so the granddaughter can get in. He won't answer the phone if she rings from outside… I'll have to call back*. *After packing my suitcase, I head out the door… He goes to lock the door himself because he doesn't trust me to lock it*. Every time before I leave, I think: maybe this is the last time I can leave him for a week…

#### 3.2.3 The bond in care

**Figure 23:**
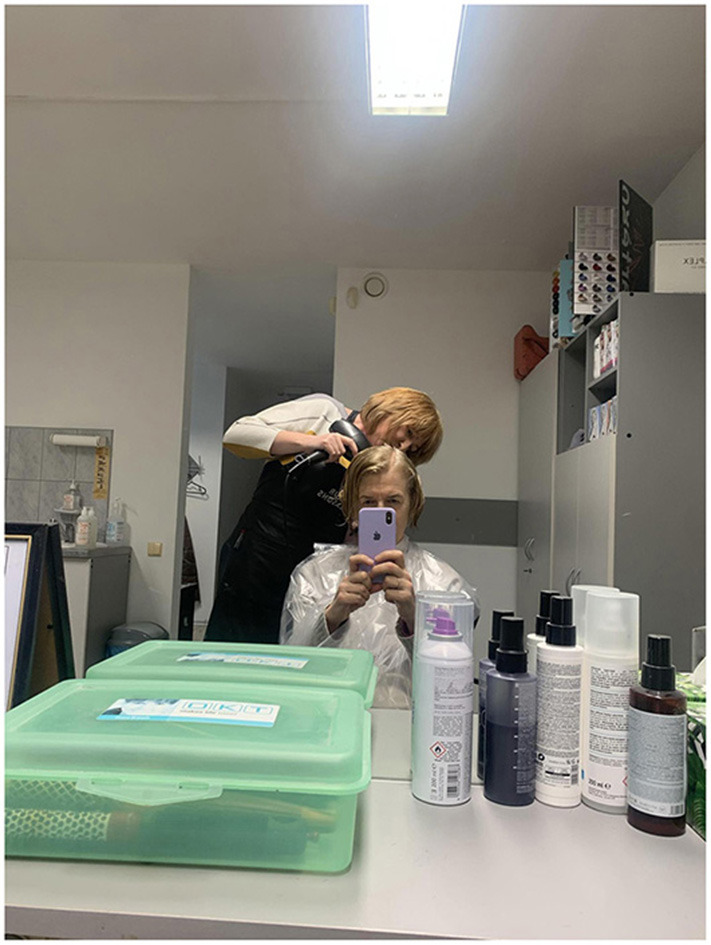
Zaneta, “*Small Joys”* When something is wrong with my mother, I feel bad too and don't want to do anything. A visit to the hairdresser lifts my mood. It's a time to be with myself. Then I feel like a different person. I invite the hairdresser to our home for my mother too. She enjoys it, and I feel joy seeing her looking beautiful… One doctor says: “They drag you along with them.” Does this apply to everyone? How can you cope? *When I feel good, I find time for myself*.

**Figure 24:**
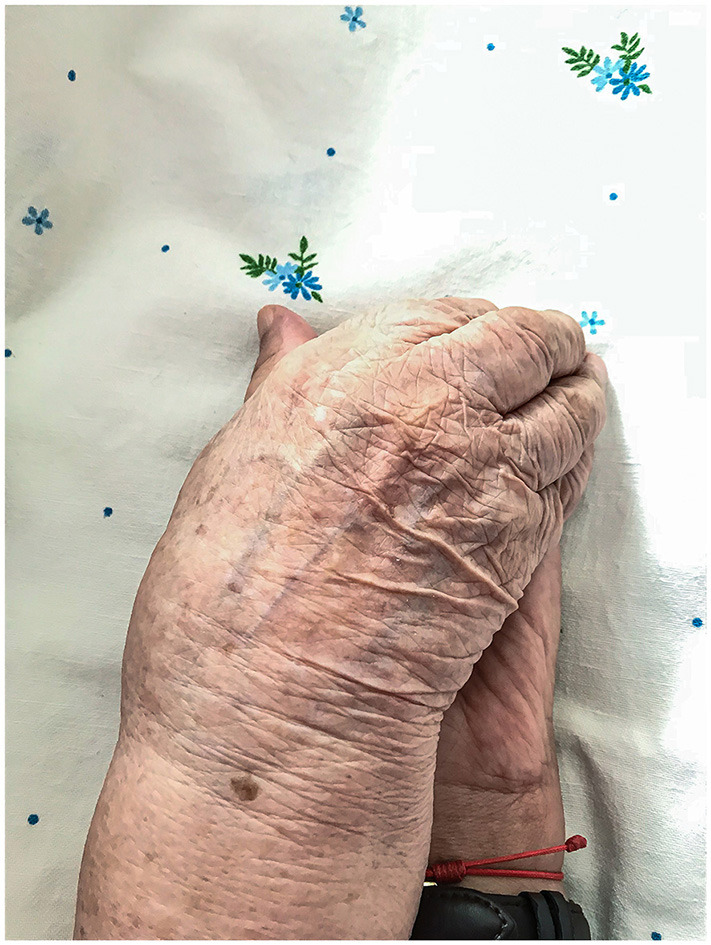
Roma, “*My Little Child”* Despite all the challenges, all the difficulties, the anger and everything else. Mother's love remains. You feel that love when she says, “My little child.” She says it rarely. Maybe once a month. But it feels so good, so good, because you feel like a child again… Without a mother, without parents, you are no longer a child. She rarely wants to show love… more often she interacts with her little dog. But sometimes she takes my hand, places her hand on mine, wants to hug me. Then that love appears, that feeling, that safety. It's not only me who needs a mother. It's not only me who wants to feel like that child, that daughter. She needs protection, so that she feels safe… Once the doctor asked her: “Who accompanied you?” “I came here with my mother,”– she said. Consciously she doesn't grasp it… Not with her mind, but with her heart, she feels safe. For her, home is where I am. Where her dog and cat are. *Not where she lived her life, but where I am*.

**Figure 25:**
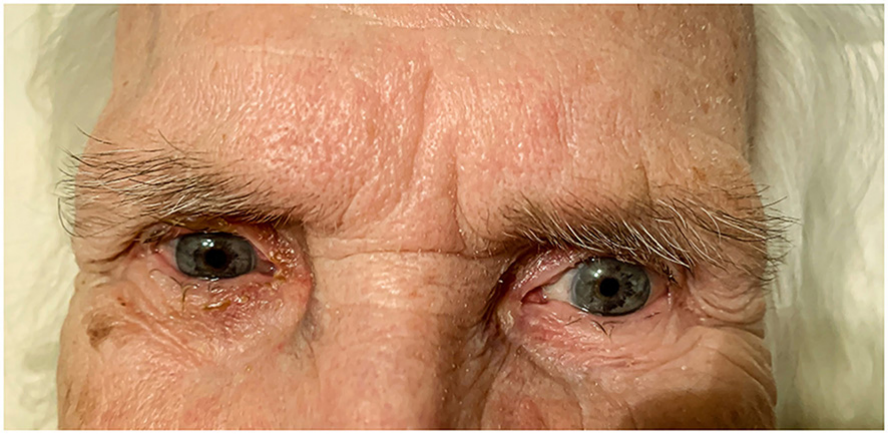
Žaneta, “*My Mother's Eyes”* *Sometimes her eyes sparkle, sometimes they don't*. *Her eyes radiated a light that I wanted to capture in a photograph*. *Sometimes her eyes are extraordinary*. *As she is lying down, they shine like little stars*. *You can see the soul, the kindness*. *Wisdom, life experience*. *Energy from nature*. *A mirror of the soul*. So much pain in there… *Life is very painful*. *You're trapped in your room, unable to move anywhere*. They sparkle especially when she sees her grandchild, when she sees strangers… *With me, they rarely sparkle*. And when they do, I feel so happy… *Like a moment of illumination—the grandmother becomes the grandmother she once was*. Then I think, all the energy I give isn't in vain… *In other moments she's gone, nothing sparkles*. “*Where did you go?” I ask her*. *She stares, staring*. When she leaves, she is behind that “barrier,” veil, fog… *Something closes*.

**Figure 26:**
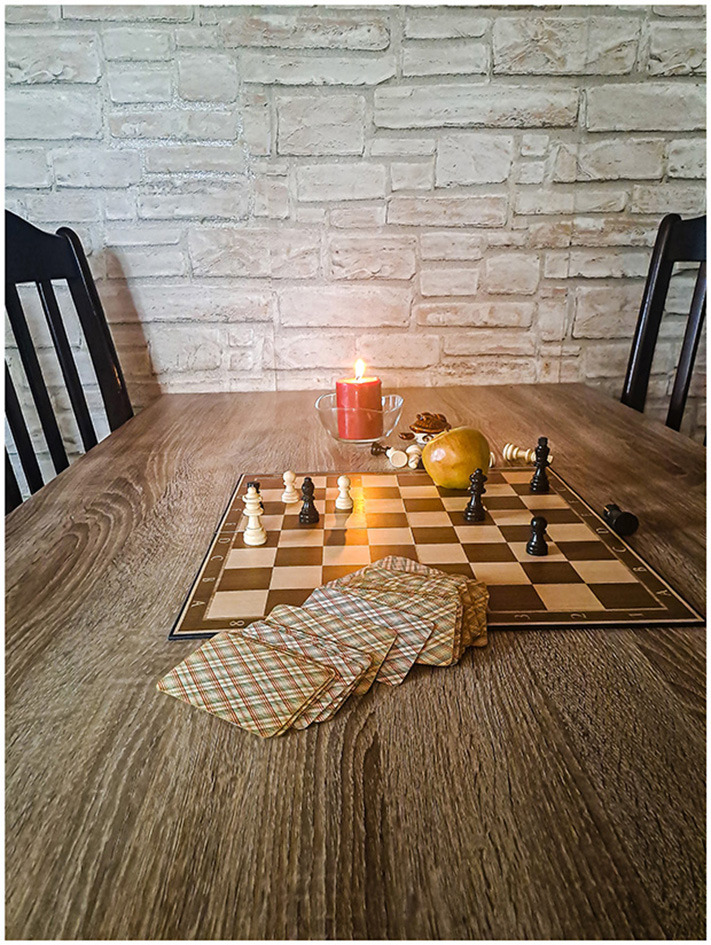
Rima, “*Waiting for Freedom”* *A friend (who knows me well) gave a name to this photograph… You nibble a little apple. Keep your mind busy and wait for freedom. Wait, don't focus on the problems. Even if you are stuck between four walls and can't go anywhere, a break from daily life is a choice*.

**Figure 27:**
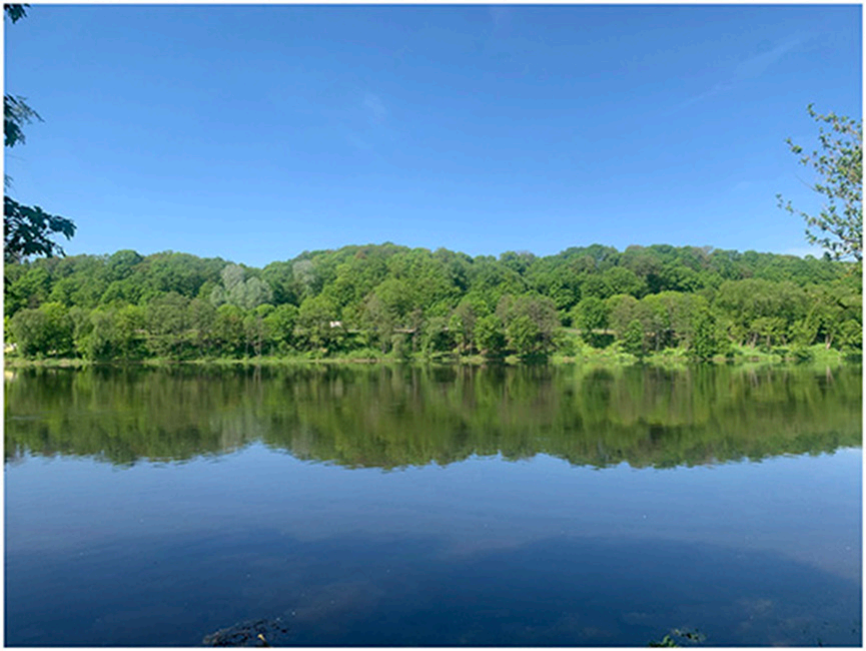
Žaneta, “*Reflections of Life. Sometimes Visible, Sometimes Not”* *I often photograph the Nemunas River. Sometimes the water reflects a perfect image. Sometimes you have to pretend that everything is fine. No one can see your inner life. Those who don't know how you live won't understand. Others don't care that you're tired. When I get tired, I feel different and more anxious*. *Anxiety that my mother won't fall, that she won't hurt herself, that she won't get out of bed, that she won't pull off her diapers, that she won't harm herself… I have to control myself, so that when I leave, I don't think something bad will happen. I have to believe that everything will be fine, that I'll find things as I left them*. *I tell myself everything will be fine. I have to pretend I'm calm if she wakes up with someone else, without me*. *She was in the hospital for a week. I ran around more than when she was at home… I visit before work. I visit after. She tears everything apart, won't let them take blood. The nurses say: “Come, she won't listen to us.” She only listens to me*. *There's so much anxiety… If I'm worried, I can't relax in the hair salon or anywhere else*. *I have to learn to be here and now*.

Also family care partners pointed out the theme of the bond in care (Figures 23–27).

#### 3.2.4 The role of supportive-social environment

**Figure 28:**
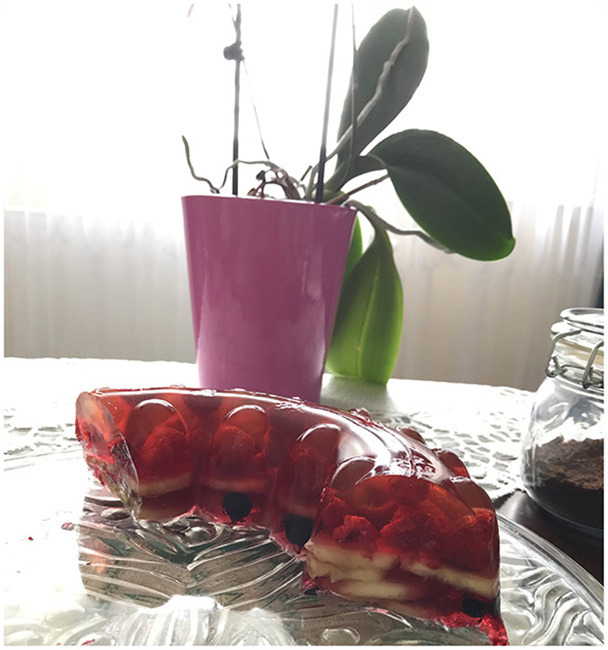
Roma, “*Friends Remained Friends”* *We went to her old home. A neighbor, her peer, came over. We drank coffee, tea, and talked*. *In her current state, my mother cannot meet with those neighbors every day, but they come, visit, and interact. Maybe they do it for my sake, but it is also pleasant for my mother. She smiles, sits contentedly… She has always been the soul of the company… Perhaps the friendship with her friends has lasted because of her kindness*.

**Figure 29:**
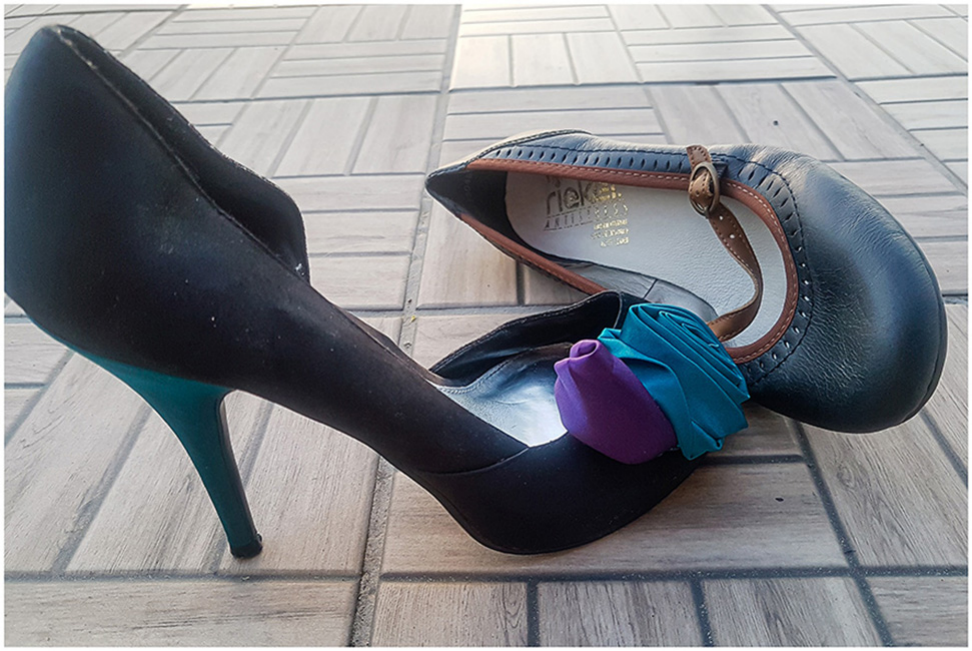
Jovita, “*Passing”* *The intersection of weary old age and youth*. *Children must provide care. Grandchildren help with caregiving*. *The hardest part is accepting the situation as it is. The hardest part is accepting that the person is leaving*. Acceptance has been ongoing for two years… *I cannot come to terms with the fact that I cannot change anything*. It is hard to lose hope, to only watch and see it fade. It is hard to watch… I started talking with my children about what it would be like if it happened to me…

**Figure 30:**
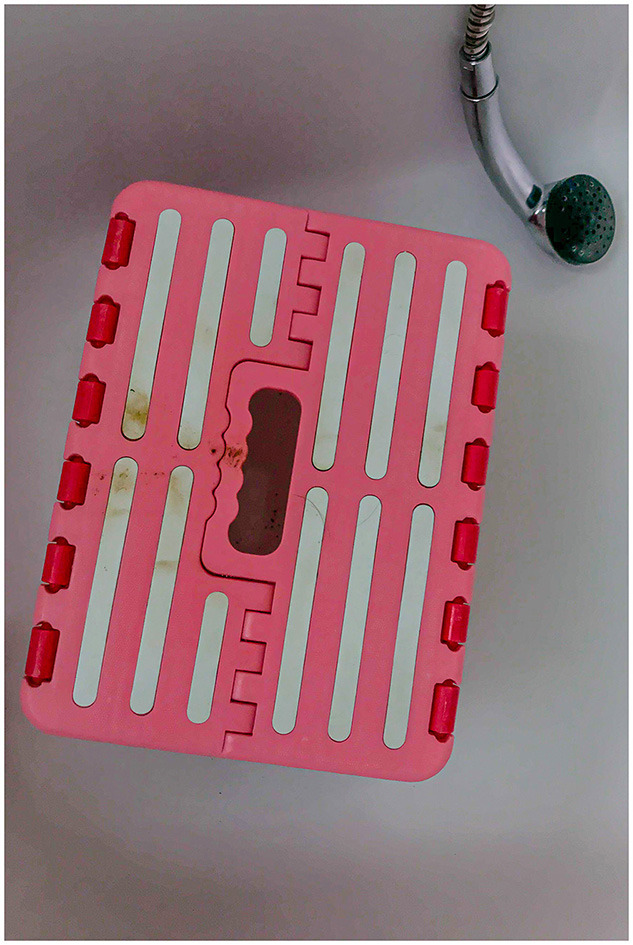
Roma, “*And again”* When Mom stopped walking, I went to the doctor to ask for specialists to come and help me. A woman arrived who washed her. And how does she manage to get Mom into the bathtub? She's smaller than me! But in no time—Mom was in the tub. Washing her was a huge problem for me. And it went on every day. She weighs over 80 kg. After every wash, I felt like a sick person myself—unable to even turn in bed or do anything… I did everything just to hurt less. *This problem was solved once we received the services*.

**Figure 31:**
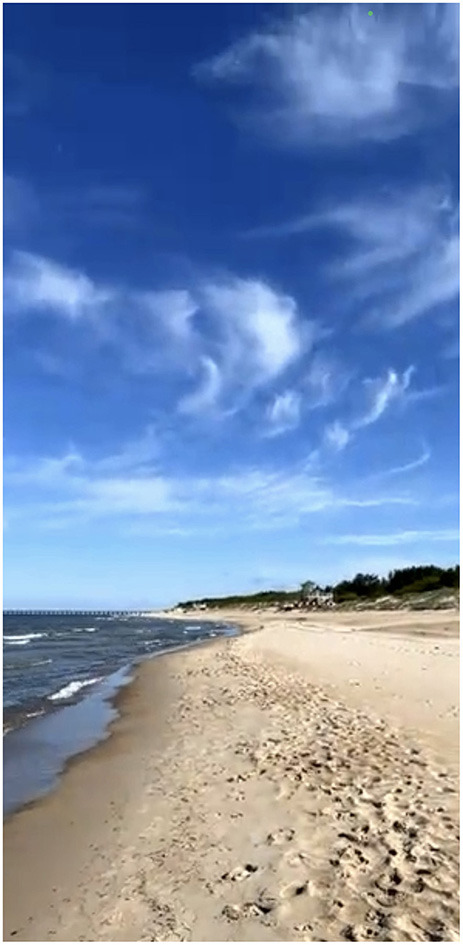
Žaneta, “*Endless Desire for the Sea…”* The photo was taken during a video call. A friend called from Palanga: “Look where I've ended up!” shows it, walks around with the camera… It's nice that she shares it, but I myself need to be by the sea. I went a year ago. I arrived, breathed in the air, sat for a while, listened to what the sea was saying… *To go to the sea at this stage of life, you really have to want it. You go just to spend a few hours, but it's worth it because being there is so important. You can't suddenly allow yourself this kind of desire. Only when the week is freer… I have a team that I need to organize so that everyone can spend some time without getting exhausted. A woman comes, my son comes*. Here, only I can spend the whole day with my grandmother. Sometimes my son says: “You go, I'll stay.” That woman is also very kind. I'd like to take her too, but I can't—who would stay with the grandmother? She helps me a lot. She asks: “Is it hard for me to spend 3 hours with the grandmother?” *She comes, stays, talks, sings, feeds… I want to show my gratitude in more than just financial terms. I feel very limited. But I have to figure out how to manage it without involving other people too much or burdening them*.

**Figure 32:**
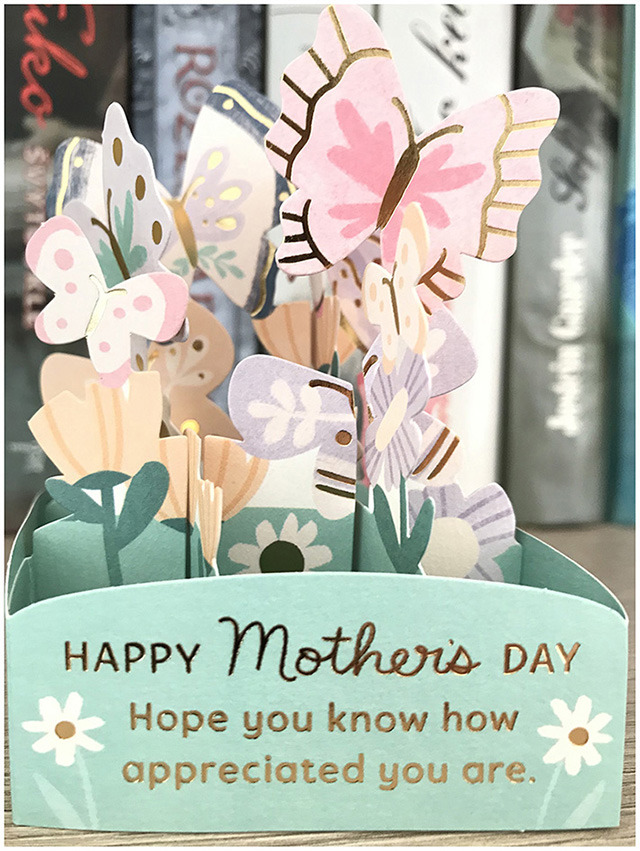
Roma, “*Motivation”* *For every mother, the greatest support comes from her children, and my daughter-in-law is like a second daughter to me. They support me. I know I am not alone… I am loved, not forgotten. When things get really hard, when the routine wears me down, when it hurts, they are my motivation to keep going, to participate, to communicate, to not close myself off, to not hide the fact that my mother has Alzheimer's*. “*Mom, you're always smiling, you never say you feel bad. Maybe that's why your grandson is such a little jokester.”* *My son lives in England. So does my grandson. I received this card when he was six months old. I had seen him only once at Christmas, and now at his christening, when they visited. I wish I could visit my grandson whenever I want*. My ability to do that depends on who is looking after my mother… Everyone understands, everyone promises—we'll let you go. But when it comes down to it, only the neighbor helps. Leaving my mother in a care home is impossible. Maybe there would be a place if the diagnosis were not Alzheimer's or dementia. Some would accept her for a week, but as soon as they learn the diagnosis, they say “no.” *A nursing hospital—very long procedure… You get a referral, but it's unclear when your turn will come, and you have to plan… Sometimes a spot opens the next day, but that doesn't help. My son has to get vacation time, tickets need to be bought*.

Moreover, a theme of the role of supportive-social environment was also identified by the participants (Figures 28–32).

## 4 Discussion

### 4.1 Focusing on care partner well-being

All of the participants in this study were women (*n* = 10). According to the literature, women tend to have higher levels of anxiety than men due to biological and sociocultural factors (34). Women experience higher disability-adjusted life years and mortality due to dementia, and they also provide 70% of care hours for people living with dementia (2). Additionally, informal caregivers with full-time jobs reported better mental health than unemployed or marginally employed caregivers. The significant interaction term for full-time and part-time employment indicated a moderating effect of net household income on the association between employment status and mental health. Employment appears to be a relevant protective factor for informal caregivers' mental health. However, if informal caregivers are not employed, a low net household income may further restrict their mental health (35). In our study, half of the caregivers were unemployed (*n* = 5). Some caregivers stated that they found it difficult to balance the roles of caregiver and worker, or that they had to leave work due to caregiving responsibilities. Therefore, welfare policy structures must be developed to reduce the negative financial consequences for informal caregivers and enable them to achieve work-life-care balance (35). Greater financial support may have beneficial effects in protecting caregivers' well-being (36). Many studies have shown that the complex and progressive nature of dementia-related family caring is exhausting, and is associated with psychological harm and a loss of well-being (37). Caregivers report worse psychological and physical health (38–40). Thus, it is important to support the caregivers' well-being so that they, in turn, can better care for individuals living with dementia.

### 4.2 Accessibility of Photovoice

Wang et al. (38), in his research, suggested that caregiver interventions improves quality-of-life measures and reduce caregiver stress. Addressing caregivers' well-being is essential for enhancing overall societal health and productivity (38). The five ways to well-being—connect, be active, take notice, keep learning, and give—as indicated by the New Economics Foundation (41), were active components in this study. The process of Photovoice offers a gentle way to engage with people with diverse needs and abilities, supports them in exploring their lives and experiences, and facilitates communication through photography and conversations with others (30). According to Wang (27), Photovoice is a participatory action research strategy that may offer unique contributions to women's health.

Application of the Photovoice method considers flexibility to suit the situation and needs of the participant. It is important to acknowledge that the person's situation affects their participation, yet the guiding principle is to support the participant's confidence and encourage them to represent themselves, their views, and ideas to others. The professional facilitation is very important to ensure the quality of the process and its ethics (30). According to Casey et al. (42), the recruitment of people living with dementia and their caregivers was a significant challenge and led to considerable delays in the initiation and implementation of the intervention. In this research, to ensure caregiver attendance at meetings, participants were offered a schedule of on-site workshops designed to match their availability. For example, we implemented a careful registration process to select candidates who demonstrated strong interest and the capacity to complete the study. We also provided a timetable of study activities tailored to participants' needs and availability, such as delivering workshops on Saturday mornings and offering financial support for attending on-site meetings. Close communication with participants was maintained through the study, including a phone calls and one home visit, to foster a stronger relationships, support completion of creative task, and address any questions.

### 4.3 Care relationships: adaptation, self-reflection, continuous learning, acceptance and kindness

Previous research highlights that transitioning into the role of family caregiver entails major individual consequences, often leading to intertwined changed roles and a reshaping of the caregiver's life course (21). Undertaking the role of a family caregiver for a person living with dementia frequently leads to a new, unexpected, and unwanted life situation. As dementia progresses, caregivers must adjust to new roles, tasks, and responsibilities. Adapting to this situation is an act of “becoming,” which involves envisioning a new kind of life for themselves as a family caregiver—that is, becoming a different kind of person due to the changed life situation (21).

In this study, care partners of people living with dementia often found themselves in challenging situations that resulted in their reactivity toward the behaviors of a person they care for. Navigating these experiences becomes a journey which entails reflecting on what has happened, learning new ways to engage, finding acceptance and becoming kinder to a person living with dementia, while also forgiving themselves:

“*You search the whole house for hidden objects, collect food leftovers from the bed, take toilet fresheners out of the fridge, persuade her to eat, to wash, and maybe for the 10th time you answer the same question again… I lose control… I get angry… But how could you get angry? Then guilt creeps in. She's your mother! You're healthy, and she is the one with the illness. I understand that it's the disease, but sometimes it feels like she's doing it on purpose… Then comes anger at myself. How could you? You cry. The greater the anger, the deeper the self-blame. I tell myself: I really won't get angry again, I really won't scold her. But then it's the 10th time… You can never predict when and what will happen again.”* (Jovita, Figure 1)

The acceptance of the constant changes in the health of a person living with dementia is an ongoing process. The participants express that navigating this process requires care partner to be continuously alert and anticipatory of things that may bring discomfort, harm or of shame. The carers acknowledge that the experience is made more difficult by the lack of awareness about dementia, as well as by feelings of helplessness and lack of control over how dementia affects the person they care for:

“*The scariest part is that others have no idea this is an illness. They might laugh, they might insult, God forbid they might hurt her. I am afraid that mum could get hurt. It's hard to accept. I try to block it out, not to think, not to analyze. It is what it is. I wish it weren't so. When I learn to accept, then I won't pay attention anymore*.” (Vaida, Figure 2)

“*… Children must provide care. Grandchildren help with caregiving. The hardest part is accepting the situation as it is. The hardest part is accepting that the person is leaving. Acceptance has been ongoing for two years… I cannot come to terms with the fact that I cannot change anything. It is hard to lose hope, to only watch and see it fade. It is hard to watch….”* (Jovita, Figure 29)

“*It seems like everything is fine, but it's not. When you don't know what to say or what to do. It's sad that it happened this way. It's sad that this happened to my mother. Sometimes I feel sorry for myself. But I feel most sorry for my mother. She is 65, could be enjoying life, living fully. Now she finds joy only when she feels safe and calm. She is happy when I give her something to eat… She wants to go somewhere. But I can't take her… Inside—a tornado. She grabs and is swept away, can't escape. … It irritates you, but you can't change anything. Time heals, working on yourself helps. I am patient—as much as one can still be*.**” **(Vaida, Figure 4)

“*… She asks what day it is. And this happens a hundred times. I tell her the day, and she calms down. You have to accept the person as they are, not try to change their thinking. It took me more than a year to accept this… Earlier, I didn't listen and wanted things my way: you need to wash, you need to tidy up, you need to be here and there. I have to accept her questioning, that she will keep asking …”* (Vitalija, Figure 7)

The care partners notice that their life in the caregiving role is disconnected from life outside their home. The perceived lack of concern from others about their situation contributes to feelings of loneliness, isolation, and despair:

“*In the past, pigeons were domestic animals—cared for and loved. When there they were no longer needed, we abandoned them. Members of society often look down on those they consider useless. A society is only as strong as its most vulnerable members. Disability, illness, loss of function, and so on are part of life*.” (Aušrinė, Figure 11)

“*Now my mother's teeth hurt. Normally, when you're in pain, you can go without waiting… But when an old woman arrives, unable to walk, they take a look and say: ‘There's no inflammation,' and send her away. But she's in pain. My mother is in pain, she doesn't eat. My heart breaks from that pain, because I feel it all. No one cares. If it's not your mother* – *no one cares.”* (Žaneta, Figure 13)

“*During holidays, she used to set the table, and her friends would come. Now her friends call me and all they talk about is the illness. They say, ‘Hang in there, if anything—you have my number.' And it sounds like, ‘Invite me to the funeral.' I'm tired of pity. I would like to say: Come, spend time together while we still can! You can't socialize over the phone, and you can't celebrate a birthday that way.”* (Jovita, Figure 12)

“*Yesterday I spoke with a relative: ‘How are you?' I said: ‘Good.' ‘How can it be good? It can't be good…' It is hard. Really hard. But who cares?*” (Žaneta, Figure 15)

### 4.4 Personal strategies for emotional awareness and attitudinal change

Care partners of people living with dementia experience anxiety, exhaustion, and isolation on a regular basis, while managing daily caregiving tasks and pretending to remain calm and composed for others. Mundane is framed by repetitive responsibilities, a lack of control, and the emotional load of unseen challenges, requiring them to constantly search for strategies to cope with the relentless demands of caregiving. Supportive to one's well-being is finding inner strength to move forwards demands, emotional awareness and determination to change in attitude toward the situation and encourage one-self when having anxiety filled racing thoughts:

“*Sometimes you have to pretend that everything is fine. No one can see your inner life. Those who don't know how you live won't understand. Others don't care that you're tired. When I get tired, I feel different and more anxious.”* (Žaneta, Figure 27)

“*The bathroom is a manifestation of what can never be finished. It is still without cabinets, without anything… without a foundation. The lack of a foundation allows disorder to accumulate. There is noise in my head. Physical, inner noise. A lack of system. I feel that everything is my responsibility. Every day, you have to do the same thing over and over. It's strange to encompass so much… Each day is a simple, domestic tragedy. Work and family. You are in a small circle that isolates you. You are what you return to every day: a limited number of people, your earned money, your work. Caregiving becomes an internal, hidden, isolating process. People lack the energy and time to be human. Sisyphus is happy because it's not worse than it is. He has already accepted the absurdity of his situation and can roll the stone with satisfaction. The only way to survive is to live with a positive attitude. I chose not to be sad and to move forward.”* (Aušrinė, Figure 3)

“*Anxiety that my mother won't fall, that she won't hurt herself, that she won't get out of bed, that she won't pull off her diapers, that she won't harm herself… I have to control myself, so that when I leave, I don't think something bad will happen. I have to believe that everything will be fine, that I'll find things as I left them. I tell myself everything will be fine. I have to pretend I'm calm if she wakes up with someone else, without me.”* (Žaneta, Figure 27)

“*A tangle of illness, institutions, trips, unfinished tasks. It is what it is. What I can no longer do, I no longer need to. I had plans to grow old gracefully, to sail on the Nile. I thought, like everyone else: I'll retire, I'll travel, I'll go, I'll sail… You plan one way, life takes another. I am like that little bird without a nest. Now there is only one branch. Not much to bring along, only I decide… Everyone can listen, but you decide what to do yourself. Many say: ‘Don't worry—he's sick.' If I just want to vent, I hear again: ‘Don't worry.' If you haven't experienced it, haven't lived alongside—you won't understand. No one will understand, whether from a joyful story or a sad one. Which branch—thinner or thicker—should I squeeze through? There is a choice of which branch to sing on—there are equal, thin, bending ones. I still have a branch.”* (Janina, Figure 8)

“*She was in the hospital for a week. I ran around more than when she was at home… I visit before work. I visit after. She tears everything apart, won't let them take blood. The nurses say: ‘Come, she won't listen to us.' She only listens to me. There's so much anxiety… If I'm worried, I can't relax in the hair salon or anywhere else. I have to learn to be here and now*.” (Žaneta, Figure 27)

### 4.5 Joy and happiness in here and now interactions

Despite the negative impacts of caring for people living with dementia, caregivers perceived their role as associated with blessings, deriving positive meaning and fulfillment from their caregiving journey (43). This study also supports the findings of Quinn (44), indicating that at times, caregiver families experience positive effects of care, such as a stronger connection with their loved one and emotional satisfaction in providing necessary support.

In our study, family care partners have found that mindful presence allows to experience joy and happiness in the small moments of interaction with the person living with dementia—they brighten their day and emerge as a source of motivation:

“*Sometimes her eyes are extraordinary. As she is lying down, they shine like little stars. You can see the soul, the kindness. Wisdom, life experience. Energy from nature. A mirror of the soul. So much pain in there… Life is very painful. You're trapped in your room, unable to move anywhere. They sparkle especially when she sees her grandchild, when she sees strangers… With me, they rarely sparkle. And when they do, I feel so happy… Like a moment of illumination—the grandmother becomes the grandmother she once was. Then I think, all the energy I give isn't in vain… In other moments she's gone, nothing sparkles. ‘Where did you go?' I ask her. She stares, staring. When she leaves, she is behind that ‘barrier,' veil, fog… Something closes.”* (Žaneta, Figure 25)

The shared connection with a person living with dementia provides caregivers with a sense of safety, love, and a meaningful human connection:

“*Despite all the challenges, all the difficulties, the anger and everything else. Mother's love remains. You feel that love when she says, ‘my little child.' She says it rarely. Maybe once a month. But it feels so good, so good, because you feel like a child again… But sometimes she takes my hand, places her hand on mine, wants to hug me. Then that love appears, that feeling, that safety. …”* (Roma, Figure 24)

“*Her bed, her room—the safest place. She feels best in her own bed… She sits. Feels safe. Mom always watched when the sun rises and sets. ‘The sun sets at 9, go home.' At first, I thought she wanted to send me away, but now I understand—it's her care: the child leaves, so that you are safe.”* (Vitalija, Figure 7)

“*Everything comes to an end… This isn't about death. Everything will end eventually. I have to live through the present moment. Every task has its ending, every moment, every period. I cherish each day: that my mother is here, that she smiles when everything is going well.”* (Žaneta, Figure 6)

### 4.6 Nature for well-being

Spending time in nature helps caregivers relax, restore their energy and support their well-being:

“*The thick lindens planted by my grandfather protect from the sun, you can hide when it's hot. I wanted those birdhouses so I could watch how the little birds settle in. I waited for the starling to make its home. Now I watch how it flies, how it feeds. It is relaxation, when you feel you can't go on anymore*.” (Vitalija, Figure 16)

“*Nature accepts you as you are. It helps you feel the ground beneath your feet, to collect yourself together so you can move forward: to go back to work, to take care of your family. When I first learned about the illness, there was a stage of mourning. Now—the caregiving stage, or ‘rollercoaster.' There were bad days and good days. Now—unpredictability. Good minutes and bad minutes. Constantly stepping out of the comfort zone… It is still hard to accept that this is my life. What could I have done differently so this wouldn't have happened to me?”* (Ingrida, Figure 5)

Even simple moments of going outside to breathe fresh air provide care partner with a chance of brief rest during the demanding daily tasks:

“*Lying down to rest legs after moving around, after walking through the house. When he rests, I try to go outside or sit in the fresh air…”* (Rima, Figure 19)

Activities such as walking by the river and taking photographs of natural surroundings offer a moment for creativity and relaxation:

“*I often photograph the Nemunas River. Sometimes the water reflects a perfect image.”* (Žaneta, Figure 27)

Hiking and various other activities in nature help to manage mental and physical exhaustion and find relaxation. Better balancing rest and workload provides an insight into the interconnectedness of caregivers' health and well-being and the quality of care provide:

“*Nature, Where You Recover This is my place of calm. When I start getting irritated, when I'm overtired. I've already said it, I've already written it, but the questions don't stop… When I see I can't hold myself back anymore, I go out to the little pond and walk around it about six times. Now it happens less often… before, I was always unrested. I wanted to do too many things. I reduced my workload. I try not to come when I'm overtired, so I won't be angry.”* (Vitalija, Figure 21)

### 4.7 Healthy eating at home and out, visiting a hairdresser or going on a short break

Maintaining a healthier diet, along with other self-care practices, helps to sustain one's well-being and resilience while coping with the demands of daily caregiving:

“*The greenery—so that I can be stronger. This is my daily joy. It's a piece of strength that I receive from nature. Those batteries run out… Who will recharge them? The load at work and here is similar. But here it's my problem. My relatives say: ‘How hard it must be for you, how hard it must be.' It's not really hard, not like that. Sometimes it's hard—you rest and then go on…It's very hard when I feel unwell. When I feel healthy, strong, then it isn't hard for me. …”* (Žaneta, Figure 15)

Occasional visit to a restaurant with another family member is experienced as a treat—it is a rare possibility for a caregiver to take a break from their responsibilities, to enjoy time together and to relax:

“*My sister and I. An emotional evening. Food. Time together. One of those days when we allowed ourselves to go out. Not for chores, just for the sake of it. It was a choice. We do most of the caregiving, and if we're not home, we get very anxious… This time we decided to treat ourselves to a good meal. It was incredibly delicious. It's great when someone else makes your food and you don't have to worry about cooking. I prepare food for my sister and grandmother. My father cooks in the evenings. At home, we don't eat together at the same table.”* (Aušrinė, Figure 20)

Visiting a hairdresser provides a family care partner with a brief escape from their routine, providing an opportunity to recharge, experience a glimpse of joy and “normalcy” associated with life outside:

“*When something is wrong with my mother, I feel bad too and don't want to do anything. A visit to the hairdresser lifts my mood. It's a time to be with myself. Then I feel like a different person.”* (Žaneta, Figure 23)

She also wished to share this joy with her mother living with dementia. Recognizing that seeing another person happy bring her a sense of happiness and fulfillment:

“*… I invite the hairdresser to our home for my grandmother too. She enjoys it, and I feel joy seeing her looking beautiful…”* (Žaneta, Figure 23)

Occasionally going on a short vacation allows caregivers to recharge. However, it requires thorough planning and coordination to ensure the well-being of the person they care for:

“*Every year I go to a sanatorium twice, to recharge myself. For a week, no longer. Before the trip, I have to start announcing the day I'll leave, at least a few weeks in advance. And I say it every day. When only a few days are left, I start preparing… I begin explaining that he'll have to get his own breakfast that my granddaughter will bring warm lunch. I ask that he doesn't leave the key in the lock so the granddaughter can get in. He won't answer the phone if she rings from outside… I'll have to call back. After packing my suitcase, I head out the door… He goes to lock the door himself because he doesn't trust me to lock it. Every time before I leave, I think: maybe this is the last time I can leave him for a week…”* (Janina, Figure 22)

When caregivers feel mentally well, they are more able to prioritize and take care of their own well-being:

“*… One doctor says: ‘They drag you along with them.' Does this apply to everyone? How can you cope? When I feel good, I find time for myself*.” (Žaneta, Figure 23)

### 4.8 Improving interior of living place or attending an arts activity

A woman who moved in to provide care for her mother, who has dementia, decided to make changes to her mother's apartment—to renovate the balcony in order to let in more natural light, hoping that the brighter, more open space would ameliorate how she feels in this home:

“*Longing for Light… The handyman came. He asked: ‘What do you want me to fix?' ‘The balcony!' These houses belong to my mother. The balcony had been glazed to protect us from the sun and the cold. That's why it felt gloomy, dark. A feeling of being trapped. Along with it—loneliness, routine, anxiety… I wanted to let light in—life goes on. When that feeling came to me, everything just exploded. I couldn't find a way to have access to nature, freedom, fresh air, light. You have to create it yourself, make it happen so that there is light. When the balcony was opened, I was so happy. I had never felt that way in my life. I feel stronger because of what I did. ‘Why do you need that balcony?' someone asked. And I just suddenly went ahead and did it. And I felt so good that I just did it.”* (Žaneta, Figure 18)

Another care partner, who looked after her grandmother, participated in an arts activity, which she found helpful for improving her well-being. She noted, that arts could be beneficial for the person living with dementia. However, she was uncertain whether she could afford to attend regularly, as there was no one available to help assist with caregiving while she was away:

“*Forced Slowing Down After a long time, I left home to meet a person I hadn't seen in a while. It was very difficult to draw with ink. The more tense you feel, the harder it becomes. After a while, you realize that you have no choice—you must relax, otherwise you can't control the brush. Life is tense, fast. I have to live for two people, feeling responsibility for myself and for another. But it's impossible to rest for two people. Rest is one step forward. Returning to everyday life is two steps back. … Artistic activity is also important for the person being cared for. My dilemma: can I make time for it? Other people perhaps cannot allow themselves that at all*.” (Aušrinė, Figure 17)

### 4.9 Emotional support, lifelong friendships and strong family relationships

The author's findings suggest that a well-functioning family may buffer the negative impact of rigid caregiving beliefs, particularly for daughters. Their results highlight the importance of interventions aimed at improving family dynamics to enhance caregivers' well-being (45).

Our study indicated that, at times when one can't easily leave their home, even a small gesture or warm word received from a close friend or family member can serve as a source of emotional support and inspiration, offering a brief moment of agency and relief from the constraints of daily life:

“*Waiting for Freedom. A friend (who knows me well) gave a name to this photograph… You nibble a little apple. Keep your mind busy and wait for freedom. Wait, don't focus on the problems. Even if you are stuck between four walls and can't go anywhere, a break from daily life is a choice.”* (Rima, Figure 26)

“*For every mother, the greatest support comes from her children, and my daughter-in-law is like a second daughter to me. They support me. I know I am not alone… I am loved, not forgotten. When things get really hard, when the routine wears me down, when it hurts, they are my motivation to keep going, to participate, to communicate, to not close myself off, to not hide the fact that my mother has Alzheimer's*.” (Roma, Figure 32)

“*I have to block my thoughts, not analyze, not dig. Hide thoughts and emotions. Suppress memories of what she used to be, so it won't be hard. Hide all of this from myself and others. I must not talk, complain, or tell. Those who haven't experienced it won't understand. I ‘withdraw' in front of those I can. The only ones I can talk to are my brother and his wife. And the children! They are wonderful. With them I have the same kind of bond I had with my mother*.” (Vaida, Figure 9)

Social isolation is an important risk factor for dementia and caregiver well-being (2). Therefore, interventions aimed at reducing loneliness (46) and social isolation (47) among caregivers could reduce their risk and support their health and well-being.

In our study, one caregiver emphasized the benefits of friendships and socializing both for her mother and herself:

“*We went to her old home. A neighbor, her peer, came over. We drank coffee, tea, and talked. In her current state, my mother cannot meet with those neighbors every day, but they come, visit, and interact. Maybe they do it for my sake, but it is also pleasant for my mother. She smiles, sits contentedly… She has always been the soul of the company… Perhaps the friendship with her friends has lasted because of her kindness.”* (Roma, Figure 28)

The caregiving situation also prompted another caregiver to discuss aspects related to her own future health and well-being with her children. The participants stressed the importance to talk with their children about the possibility that one of the partners will have dementia in the future, and that the children should know their parents' wishes in terms of caring for them:

“*I started talking with my children about what it would be like if it happened to me…*” (Jovita, Figure 29)

### 4.10 Taking leadership and focusing on self-care

The caregivers emphasized problems in the quality of general dementia care facilities. To feel less stressed, they chose to care for their loved ones themselves and to accept the current limitations of the national health and social care systems:

“*My mother is aging, but to me her beauty doesn't change. She can no longer walk, but she is still beautiful to me. The older generation of mothers never thought about preparing for life in a nursing home. They never thought about how not to burden their children—the idea was, we will live together and care for one another. Maybe now it's considered ‘beneath you' to care for your parents? Doctors and acquaintances have told me: ‘Why do you exhaust yourself so much? You can hand her over—then just come and visit.' Those who provide care at home do need rest. But can I place her in a medical facility and feel at peace? Right now, I cannot. I don't have the trust that everything will be fine: that her diapers will be changed as often as needed, that she will be offered water (since she doesn't ask when she's thirsty—sometimes she drinks, sometimes she doesn't, so you have to offer often), and so on. I fear that my mother might be harmed… I see how many people working in the care system have become numb to the fact that this is a life—a life worthy of respect. Compassion is lacking. And you can't fight the system.”* (Žaneta, Figure 13)

“*Who could help change this situation? A fairer division of work, respect for responsibilities, and empathy. In the family, there is a lack of understanding and recognition of this syndrome. What is dementia? How does it manifest? Until that exists, I continue to worry about how the person I care for is being looked after when I'm not around*.” (Aušrinė, Figure 17)

“*Leaving my mother in a care home is impossible. Maybe there would be a place if the diagnosis were not Alzheimer's or dementia. Some would accept her for a week, but as soon as they learn the diagnosis, they say ‘no.' A nursing hospital—very long procedure… You get a referral, but it's unclear when your turn will come, and you have to plan… Sometimes a spot opens the next day, but that doesn't help. My son has to get vacation time, tickets need to be bought*.” (Roma, Figure 32)

The study found that caregivers actively sought ways to improve problematic situations and enhance their daily care experiences while also taking care of their own well-being.

One caregiver decided to consult a mental health specialist. It was useful not only for her own mental well-being, but also provided new strategies to improve the care she provides for her mother living with dementia. Her focus in care turned toward supporting and encouraging her mothers' who has dementia self-care and independency, and offer some respite for neighbors:

“*Before, I used to call the neighbor more often: she didn't know how to charge the phone or do other things. The psychologist advised: ‘Let her do it herself… so she understands. Don't do things for her.' Now I feel calmer. I need to call the neighbor less, because she manages to do many things on her own. The neighbor, seeing how I manage, said: I did myself a disservice. Taking care of my mother, I wanted to hand everything to her. Mum was bedridden, didn't walk at all. She never moved on her own…”* (Vitalija, Figure 14)

Despite disparities in available services for caregivers, there were some positive experiences. In one case, a family care partner took leadership creating a change in how services were delivered at her municipality. Although the process was challenging, the efforts proved worthwhile for her well-being:

“*I organize home help myself, persuading a private company to start providing these services in my mother's town. It turns out there is a need for such services; after me, they started providing them to others too. There is now some support from the municipality as well—they cover half the cost of the services. Caregivers don't always know their rights*.” (Jovita, Figure 10)

Another caregiver found it difficult to bathe her mother when she was no longer able to walk,

“*When Mom stopped walking, washing her was a huge problem for me. And it went on every day. She weighs over 80 kg. After every wash, I felt like a sick person myself—unable to even turn in bed or do anything… I did everything just to hurt less.”* (Roma)

when her own physical health began to decline, she decided to request assistance and was grateful to receive services from her local municipality:

“… *I went to the doctor to ask for specialists to come and help me. A woman arrived who washed her. And how does she manage to get Mom into the bathtub? She's smaller than me! But in no time—Mom was in the tub. This problem was solved once we received the services”* (Roma, Figure 30)

### 4.11 Importance of social support and supportive environment

Furthermore, participating in peer support groups offers the opportunities for sharing and exchange of the experiences, collective compassion, and problem solving, positions each caregiver as both a learner and a teacher (48). According to authors (30), the collective Photovoice practice-based process offers a more accessible space to communicate (using not only words, but also a visual language) and a bonding environment for sharing and reflection. In this study, the Photovoice practice created supportive environment and experiential learning opportunities, which provided empowering learning outcomes for informal caregivers. The space for sharing and exchanging experiences helped them to develop dementia care skills and, importantly, self-care skills, which are crucial in the role of a family care partner of a person living with dementia.

More frequent help-seeking behavior can support problem-solving and alleviate the burden of care (49). In this study, asking for a help was a key factor in maintaining one's well-being and taking a brief break:

“*Mom, you're always smiling, you never say you feel bad. Maybe that's why your grandson is such a little jokester. My son lives in England. So does my grandson. I received this card when he was six months old. I had seen him only once at Christmas, and now at his christening, when they visited. I wish I could visit my grandson whenever I want. My ability to do that depends on who is looking after my mother… everyone understands, everyone promises—we'll let you go. But when it comes down to it, only the neighbor helps.”* (Roma, Figure 32)

“*At first, two clocks were enough. Then we bought maybe five more, because something was always wrong: if it was even a minute off, it wouldn't work for her, and she would start twisting it. When she turns it, she no longer knows the time. I bought one that can be wound up. She learned how to do it. And she also gets to move her fingers. She needs two chargers, two phones, so she can make calls. The phone cords were identical, so I wrapped one in tape: red phone—red cord. She uses the phone and charges it. If something goes wrong, I call a neighbor—she goes to check that everything is okay.”* (Vitalija, Figure 7)

Even in such cases, a care partner was worried not only about her own ability to care for her mother but also about whether other providing help could do so without becoming exhausted. To make sure this does not happen, they carefully plan what happens when they briefly away—all the tasks and supports—so that, as the carer assumes, no one is overburdened:

“*Endless Desire for the Sea… The photo was taken during a video call. A friend called from Palanga: ‘Look where I've ended up!' shows it, walks around with the camera… It's nice that she shares it, but I myself need to be by the sea. I went a year ago. I arrived, breathed in the air, sat for a while, listened to what the sea was saying… To go to the sea at this stage of life, you really have to want it. You go just to spend a few hours, but it's worth it because being there is so important. You can't suddenly allow yourself this kind of desire. Only when the week is freer… I have a team that I need to organize so that everyone can spend some time without getting exhausted*.” (Žaneta, Figure 31)

“*… A woman comes, my son comes. Here, only I can spend the whole day with my mother. Sometimes my son says: ‘You go, I'll stay.' That woman is also very kind. I'd like to take her too, but I can't—who would stay with the grandmother? She helps me a lot. She asks: ‘Is it hard for me to spend 3 hours with the grandmother?' She comes, stays, talks, sings, feeds… I want to show my gratitude in more than just financial terms. I feel very limited. But I have to figure out how to manage it without involving other people too much or burdening them*.” (Žaneta, Figure 31)

## 5 Conclusions

The number of people living with dementia is increasing rapidly globally. This research question presented in this article aimed to investigate the lived experiences of what supports the well-being of family care partners of people living with dementia in a care relationship through the implementation of the Photovoice practice in Lithuania. All participants in this study were women (*n* = 10) aged from 26 to 68 years old. Among them, the majority were daughters (*n* = 8) caring for their mothers (*n* = 7). Also, half of women (*n* = 5) were unemployed, and most of them lived together with a person who has dementia (*n* = 6). None of the participants had prior experience with Photovoice or photography arts activities (*n* = 10). In conversations to discuss the photos, a number of themes related to supporting well-being emerged, such as “Reconciliation and Acceptance,” “The Aspiration to Maintain One's Inner Freedom and Well-being,” “The Bond in Care,” and “The Role of Supportive-social environment.” The family care partners of people living with dementia identified the importance of taking care of their own health and well-being, and managing the stress and fatigue they experienced. The supporting elements for their well-being, as identified by the participants, were the following:

- Enhanced learning through continuous reflective process: supported self-care awareness, encouraged communication and self-expression, dementia care skills.
- Acceptance the changes in the health status and behavior of the person living with dementia.
- Attitudinal changes toward dementia at individual, family and societal levels.
- Emotional awareness and emotional literacy.
- Mindful presence and finding joy and happiness in small moments of interaction/connection with person living with dementia gave them a sense of motivation and happiness. It was a source of safety, shared love and human connection.
- Spending time in nature to take a walk, to relax and restore energy.
- Taking photographs of natural surroundings to find moments for creativity and relaxation, and help oneself to manage mental and physical exhaustion.
- Improving rest and workload balance to gain an insight into the interconnectedness of caregivers' health and well-being and the quality of care provide.
- Maintaining a healthy diet.
- Consulting with mental health specialist to receiving practical advice how to improve care delivery and care experience.
- Making changes in your build environment: improving the interior of a living space in order to let in more natural light.
- Going out to a restaurant with a family member.
- Visiting a hairdresser or organizing a hairdresser visit at home for a person living with dementia.
- Taking a short vacation.
- Learning to ask for help and receiving assistance from support staff (such as help with hygiene for person living with dementia).
- Going to an arts activity and noticing that arts could be a beneficial tool for a person living with dementia as well.
- Maintaining friendships and socializing with other people.
- Asking for a help from others to take a break.
- The positive effect of small acts of kindness—a word or a gesture from other people may become a source of inspiration.
- Noticing domino effect: when you are feeling better mentally, there is a wish to take care of your own well-being.
- Learning to accept calmly the limitations of the national health and social care systems.
- Coming to terms with one's choice to become a care partner.
- Putting the efforts to create a change in service provision system that would serve not only for the caregiver, but likely for other people as well.

There were numerous internal struggles the participants have experienced, such as: challenges in finding someone who could come and take care of person living with dementia while they were away to improve their own well-being; feelings of guilt, grief; finding it hard to relax due to worry or anxiety about their loved ones while they were not around for different reasons (worrying whether the other carer would become exhausted or whether their loved one would be properly cared for). The caregivers highlighted problems related to the stigma surrounding dementia in their environment and society, as well as issues in the quality of dementia care, general health and social care systems, including disparities in services across municipalities and a lack of competence among care staff. To feel less stressed, they chose to take care of their loved ones themselves and to make a conscious decision to accept the current problematic state of the national care system.

In our study, the caregivers emphasized the benefits of socializing, both for themselves and for the person living with dementia, when someone visited their home. Another care partner discussed importance to talk with their children about the possibility that one of the partners will have dementia in the future, and that the children should know partner's wishes in terms of caring for them.

As the demand for dementia care is projected to grow, there is an urgent need to find ways to sustain the care provided by informal carers. Recognizing informal carers as an important part of the multidisciplinary care team, and developing efficient informal carer health and well-being promotion strategies, may counteract psychological, physical and social challenges and reduce the prevalence of stigma within the carer journey. In this study, participation in the Photovoice practice reflected “The five ways to well-being” model: participants had opportunities to connect with other caregivers, to be active while engaging in creative activity, to take notice and learn new things, and to share their experiences with others both in a group settings and in a public exhibition. The study findings suggest that the Photovoice practice methodology is a promising and valuable tool for identifying potential support systems for the health and well-being of family care partners of people living with dementia and can be applied more broadly in research and policy-making when working with vulnerable populations.

See Supplementary Data 1.2 for study limitations, strengths and further possibilities.

## Data Availability

The original contributions presented in the study are included in the article/Supplementary material, further inquiries can be directed to the corresponding author.

## Footnotes

1 Petkutė I, Karpavičiūtė S, Rėklaitienė D. *Exploring the Challenges to Well-being of Family Care Partners of People Living With Dementia Through Photovoice*. (2026) [Manuscript in preparation].
