# Supplementary material for: Using Photovoice to investigate the lived experiences of family care partners of people living with dementia: What supports their well-being in a care relationship?
Source: Front Public Health. 2026 Jul 1;14:1825358. doi: 10.3389/fpubh.2026.1825358 (PMC13370904; doi:10.3389/fpubh.2026.1825358)
Supplement: Supplementary file 1 [file Data_Sheet_1.pdf]

Supplementary Material

1 Supplementary Data

Supplementary Data 1.1. The Research Questionnaire, Administered During the Registration Process.

The research questionnaire, administered during the registration process prior to this study (Photovoice research project), consisted of the following questions:

1. What interested you in this project and made you decide to participate? (Comment):
2. Your name, surname (Write your answer)
3. Your email address (Will be used to contact you)
4. Your phone number (Will be used to contact you)
5. Where do you live? (Enter the name of the city or village)
6. How old are you? (Please, specify)
7. Are you caring for a person living with dementia? (Mark only one answer)

   Yes

   No

   Partly
8. If you answered "Partly" to the previous question, please comment:
9. What is your family relationship with the person you are caring for? (Comment)
10. What form of dementia does the person you care for have? (Mark only one answer)

    Alzheimer's disease

    Vascular dementia

    Lewy body dementia

    Frontotemporal dementia

    Unspecified dementia

    Other

    I don't know
11. If you answered "Other" to the previous question, please comment:
12. Do you live with the relative you are caring for? (Mark only one answer)

    Yes

    No
13. If you do not live together, but you visit your relative living with dementia, approximately how many times a week do you visit? (Comment)

14. How long have you been caring for a person with dementia, in years? (Mark only one answer):

    Up to 1 year

    From 1 to 5 years

    From 5 to 10 years

    10 years and more
15. If you would like, provide a comment to the previous question:
16. In addition to caring for a person living with dementia, do you work in any other workplace? (Mark only one answer):

    Yes

    No
17. If you would like, provide a comment to the previous question:
18. Do you have the following tools that may be needed to participate in the project (Please confirm) (Mark all that apply):

    I have an internet connection

    I have the ability to take photos with a smartphone or digital camera

    I have the ability to make a remote video call
19. What is your photography experience? Have you ever participated in photography art activity before? (Mark only one answer):

    Yes

    No
20. Have you ever participated in Photovoice activity before? (Mark one answer):

    Yes

    No
21. Are you willing to receive financial support to cover transportation costs for attending on-site Photovoice activities? (Mark only one answer):

    Yes

    No
22. Are you willing to receive financial support to cover the costs of a care assistant at home to attend on-site study Photovoice activities? (Mark only one answer):

    Yes

    No
23. Your additional comments or questions to us (Please, specify only):

Supplementary Data 1.2. Study Limitations, Strengths and Further possibilities.

Limitations of the study

The main limitations of the Photovoice research project were:

- A relatively small group of participants (however, according to the existing knowledge in the field, the group of 10 persons is the optimal and maximum recommended sample size, which ensures the data saturation for participation in Photovoice practice. A larger sample size would likely not generate new themes, as the themes tend to duplicate at the current sample size according to the literature and as observed in our study results).
- Lack of gender variety: all participants were female, which reflects the local and global context where the most common gender of informal care partners of people living with dementia is female.
- Participants wanted to attend more Photovoice practice / photography workshops and other arts activities after the study, but due to the limited time, budget and the impossibility of extending the project, this was not feasible.

Strengths of the study

The main strengths of the Photovoice research project were:

- This is the first and novel study to understand what supports and what challenges the well-being of family care partners of people living with dementia in a care relationship using participatory research method-Photovoice practice in Lithuania.
- This is the first cross-sectorial public exhibition to showcase the creative results of what supports and what challenges the well-being of family care partners of people living with dementia in a care relationship through the photographs to a wider audience in Lithuania. It will contribute to reducing stigma around dementia and to increasing knowledge about the importance of minimizing dementia risk factors and supporting the health and well-being of family care partners of people living with dementia among diverse audiences.
- The study findings will expand the current knowledge in the research field both locally and globally.
- This study contributes to reducing the stigma associated with cross-disciplinary, cross-sectoral, and arts for health research. Such research often involves small sample sizes and applies arts-based / qualitative and participatory research methods − specifically research that works closely with communities and empowers community members to engage as co-researchers.
- During the study, the main researcher/lead facilitator kept in close contact with participants. This ensured the success of the study – the high level of participation of care partners, motivation, etc.
- The study may inform and inspire others to apply the Photovoice practice method into their research and teaching-educational practices.
- The study has a potential to encourage others to apply arts for health activities to support the physical and mental well-being of care partners of people living with dementia in their own settings.
- The study may inform other organizations and the policy makers in developing and implementing the strategies and interventions to better support the care partners of people living with dementia.
- The study included diverse research methods to explore the research question.

- Two researchers (from health/cultural backgrounds, experienced in arts for health field) were involved in the design and implementation of the Photovoice study activities.
- One researcher completed Photovoice practice application in research training programme and had experience applying the Photovoice practice as an educational method, which expanded insight into the practical considerations, the potential, and the challenges of the method's application during the study design and implementation phases.
- The photography activities created a safe space, built mutual trust and opened new ways, and helped participants to communicate about their intimate environments to other participants and the public.
- Participants were empowered to apply the skills and knowledge gained in photography, self-care and dementia care in their daily-home environments after the study.
- Participants built a strong community during the study and have maintained contact afterward, which acted as a tool for peer support and community building.
- During the research project, the participant group enhanced their sense of well-being, increased self-esteem, and had a lifelong learning opportunity, potentially contributing to dementia risk reduction, disease prevention and improved quality of care.

Further possibilities

- Further research might investigate the potential impact of Photovoice research project on the well-being of family care partners, self-care and dementia care skills, sense of community, the communication with others, and friendships amongst the family caregivers, one year / several years after the study.
- To evaluate the impact of the exhibition, which represents the participants' creative results, on the level of knowledge about dementia and dementia-related stigma management within the attendees and wider public.
- To explore the potential impact of the Photovoice research project on the well-being of persons living with dementia.
- To evaluate the potential impact of the Photovoice research project on the quality of care and the relationships with other close family members at home.
- To observe the participants' engagement in the arts / cultural events following the study period and after the study.
- To analyze the participants' engagement in the arts activities following the study period after the study.
- To offer participants possibilities to engage in different arts activities after the study.
- At the beginning, during the introduction of the photography practice, participants could be offered an additional practical workshop to support their confidence in photography and visual literacy, encourage individual aesthetic exploration and stimulate creativity. In addition, a new workshop to support creative writing skills could be offered.
- Investigating the challenges of implementing Photovoice / arts for health research, including advocacy and dissemination of results, particularly in academic, health, and policy contexts.
- Investigating the cost-effectiveness and broader impact of implementing Photovoice / arts-for-health research on individual, societal and policy levels.
